# Influence of taxonomic resolution on the value of anthropogenic pollen indicators

**DOI:** 10.1007/s00334-021-00838-x

**Published:** 2021-05-11

**Authors:** Mara Deza-Araujo, César Morales-Molino, Marco Conedera, Gianni B. Pezzatti, Salvatore Pasta, Willy Tinner

**Affiliations:** 1 Insubric Ecosystems, Swiss Federal Institute for Forest, Snow and Landscape Research WSL, a Ramél 18, 6593 Cadenazzo, Switzerland; 2 Institute of Plant Sciences and Oeschger Centre for Climate Change Research, University of Bern, Altenbergrain 21, 3013 Bern, Switzerland; 3 Institute of Biosciences and Bioresources (IBBR), National Research Council of Italy (CNR), Corso Calatafimi 414, 90129 Palermo, Italy; 4 Department of Biology, Unit of Ecology and Evolution, University of Fribourg, Chemin du Musée 10, 1700 Fribourg, Switzerland

**Keywords:** Anthropogenic indicators, Land use, Human impact, Taxonomic resolution, Palaeoecology, Europe

## Abstract

The taxonomic resolution of palynological identification is determined by morphological criteria that are used to define pollen types. Different levels of taxonomic resolution are reached in palynology, depending on several factors such as the analyst’s expertise, the palynological school, the aim of the study, the preservation of the pollen grains, the reference collections and the microscope facilities. Previous research has suggested that attaining pollen records with high taxonomic resolution is important to reconstruct correctly past land use and human impact. This is in turn central to disentangling past human activities from other drivers of long-term vegetation dynamics such as natural disturbance or climate variability. In this study, we assess the impact of taxonomic resolution on the indicative capacity of anthropogenic pollen types. To achieve this, we attribute the pollen types of sixteen sedimentary records, located along a latitudinal gradient spanning from Switzerland to Italy, to three levels of taxonomic resolution previously proposed at the European scale. Our results show that higher taxonomic resolution improves the identification of human impact by enhancing the indicative power of important pollen indicators widely used in the research field. Our results may contribute to the improvement of palynological reconstructions of land use and human impact by identifying key pollen types whose determination requires particular attention.

**Supplementary Information:**

The online version contains supplementary material available at 10.1007/s00334-021-00838-x.

## Introduction

The classification of fossil pollen grains into taxonomic groups (i.e. pollen types) is the foundation of palynological research (Birks and Birks 1980). The precision of this identification, mostly based on morphological criteria, constrains the taxonomic resolution reached (Rull 2012). Pollen data often suffer from poor taxonomic resolution (Louys 2012), as some types are identifiable to species level but most of them only to genus or even higher taxonomic levels (e.g. family; Huntley and Webb 1988). In contrast, a sound taxonomy is one of the main requirements for palaeoecological reconstructions of environmental change (Mitchell et al. 2014), as taxonomic precision may influence the ability of pollen data to track plant and vegetation dynamics (Huntley and Webb 1988).

When reconstructing environmental changes through palaeoecology, identification to species level should always be the ultimate goal, because ecological and environmental requirements are far better defined and more precise for species than for higher taxonomic categories, and this in turn often results in more robust, accurate and reliable palaeoecological reconstructions (Birks and Birks 1980). However, taxonomic uncertainty is commonly discarded as a source of error in analogue studies, which assume that the pollen assemblages will include at least a few diagnostic taxa with high taxonomic precision (Jackson and Williams 2004). Furthermore, the taxonomic resolution attained as well as the nomenclature used vary greatly among palynologists, depending on the used palynological keys and atlases, the palynology school, the research aims, the environmental setting and the taphonomy of the pollen analysed. Moreover, some pollen analysts may not try to reach more precise identifications (e.g. to species level when possible) because they perceive that the taxonomic refinements will not substantially improve the palaeoenvironmental reconstructions (Finkelstein et al. 2006). Consequently, when dealing with datasets produced by different analysts, pollen data require a process of taxonomic harmonization where the nomenclature is standardized by identifying all the synonyms used to refer to the same morphological type (e.g. Giesecke et al. 2019). As taxonomic harmonization is increasingly common due to the growing availability of palynological datasets and big data analyses, the effects of potential information losses due to the reduction of taxonomic resolution must be well understood.

These problems are becoming particularly evident when reconstructing past land use based on anthropogenic pollen indicators (e.g. Behre 1981; Lang 1994; Deza-Araujo et al. 2020). Although these anthropogenic pollen indicators have been established based on the modern occurrence of their plant equivalents in farming contexts (Behre 1981), they are defined at different levels of taxonomic resolution depending on the ease of their identification and the authorship of the methodology (Behre 1981, 1990; Mercuri et al. 2013a; Deza-Araujo et al. 2020) (Table 1). Indeed, only a few cultivated plants can be unambiguously identified by pollen to species level (e.g. *Pisum sativum* L.; Reille 1999). Furthermore, several families or subfamilies including a number of domesticated species (e.g. Brassicaceae, Cichorioideae) bear very uniform pollen morphology and are thus commonly identified only to (sub) family level (Blackmore 1984; Beug 2004). This problem is usually circumvented by examining the synchronous occurrence of different pollen types indicative of human-modified landscapes, often involving the use of human indicator indices (e.g. OJC, OJCV, CI; Deza-Araujo et al. 2020).

**Table 1:** Original taxonomical resolution of the most used anthropogenic pollen indicators in central Europe and Mediterranean region in the literature (i.e. Behre 1981; Mercuri et al. 2013b) and in the study datasets

Anthropogenic indicator	Taxonomic resolution	Reference
*Linum usitatissimum, Vicia faba, Centaurea cyanus, Fallopia convolvulus, Scleranthus annuus, Spergula arvensis, Rumex acetosella, R. acetosa, Polygonum aviculare, P. persicaria, Trifolium repens, Plantago lanceolata, P. major, P. media, Succisa pratensis, Calluna vulgaris, Juniperus (communis), Melampyrum pratense, Pteridium aquilinum, Polypodium vulgare, Artemisia (vulgaris), Ficus carica*	Species (including synonyms)	Behre (1981, 1990)
*Secale, Hordeum, Triticum, Avena, Fagopyrum, Cannabis, Lychnis-Agrostemma, Jasione-Campanula, Urtica, Pistacia, Olea, Juglans, Castanea, Vitis*	Genus	Behre (1981); Berger et al. (2019); Mercuri et al. (2013b)
Cichorioideae, Asteroideae, Chenopodiaceae, Caryophyllaceae, Brassicaceae, Poaceae, Ranunculaceae, Cyperaceae, Apiaceae	Subfamily/Tribe/Family	Behre (1981)

Previous research has suggested that the identification of anthropogenic land use and its separation from other drivers of vegetation change such as climate would largely benefit from high taxonomic resolution of the pollen types used as human indicators (Tinner et al. 1996). Such refinement may concern the identification of introduced crops and weeds (Brun 2011; Rösch and Lechterbeck 2016). Further, achieving a good pollen taxonomic resolution is relevant to pollen richness estimates, and biodiversity change is in turn related to major land use strategies (Brun 2009; Feurdean et al. 2013). However, studies explicitly conceived to understand the effects of taxonomical resolution on anthropogenic pollen indicators are so far lacking, while other palaeoenvironmental proxies have only been rarely examined in this respect, with dissimilar results. For instance, decreasing taxonomic resolution had a relatively limited effect on reconstructed depth to water table based on testate amoebae, probably due to ecological redundancy (Mitchell et al. 2014). In contrast, chironomid-based summer air temperature reconstructions were either sensitive or not to taxonomic resolution, with the magnitude of such variation largely depending on the study site (Heiri and Lotter 2010). Regarding palynological research, studies on the effects of taxonomic precision have so far focused on biogeographical and biodiversity issues, highlighting the need for improved taxonomic resolution (Finkelstein et al. 2006) or suggesting a not always straightforward relationship between pollen richness and plant diversity (Giesecke et al. 2019; Reitalu et al. 2019). Birks (1994) also found that datasets with more detailed taxonomy notably improved quantitative reconstructions of soil pH using pollen. Similarly, low taxonomic resolution can hamper the indicative value of pollen data in palaeoclimatic reconstructions. For instance, the use of *Podocarpus* as a proxy for cool temperatures is ineffectual, as its pollen cannot be identified beyond the genus level and its constituent species have considerably variable bioclimatic preferences (Punyasena et al. 2011).

To overcome this knowledge gap and to test the effects of taxonomic resolution on land-use reconstructions, we analysed the anthropogenic pollen indicators in sixteen postglacial palynological datasets along a latitudinal gradient between Alpine summits close to the snow line and subtropical coastal Sicily at different taxonomic resolutions. This altitudinal and latitudinal gradient analysis allows the assessment of the occurrence of biogeographical differences affecting human pollen indicators, as it embraces the Mediterranean realm, where many wild relatives of (South-West Asian Neolithic) crops and weeds are native (Zohary et al. 2012) and Central Europe, where wild relatives of crops and weeds are rare (Deza-Araujo et al. 2020). Specifically, wild relatives of crops and weeds might be palynologically indistinguishable from cultivars and adventives and thus markedly affect human impact reconstructions.

The palynological datasets concerned have been produced by the same palynology school at a rather detailed and consistent taxonomy. Drawing on these records and using comprehensive harmonization tables at the European scale (Giesecke et al. 2019), we simulate datasets at lower hierarchical levels and assess the impact of this change in taxonomical resolution on the anthropogenic pollen curve and its interpretation. We use specialized literature to define anthropogenic pollen indicators at higher taxonomic resolution level than the one used routinely and to assign them to the categories of primary (crops) or secondary (introduced or opportunistic weeds) indicators. We hypothesize that reducing the taxonomic resolution will cause a decrease in the specificity and sensitivity of the pollen indicators for detecting and quantifying anthropogenic land use, with different pollen types having more or less influence on this change according to the biogeographical region. Further, we also hypothesize that lowered taxonomic levels in pollen identification will result in misleading reconstructions of past anthropogenic land use.

## Materials and methods

### Study area

**Fig. 1:**
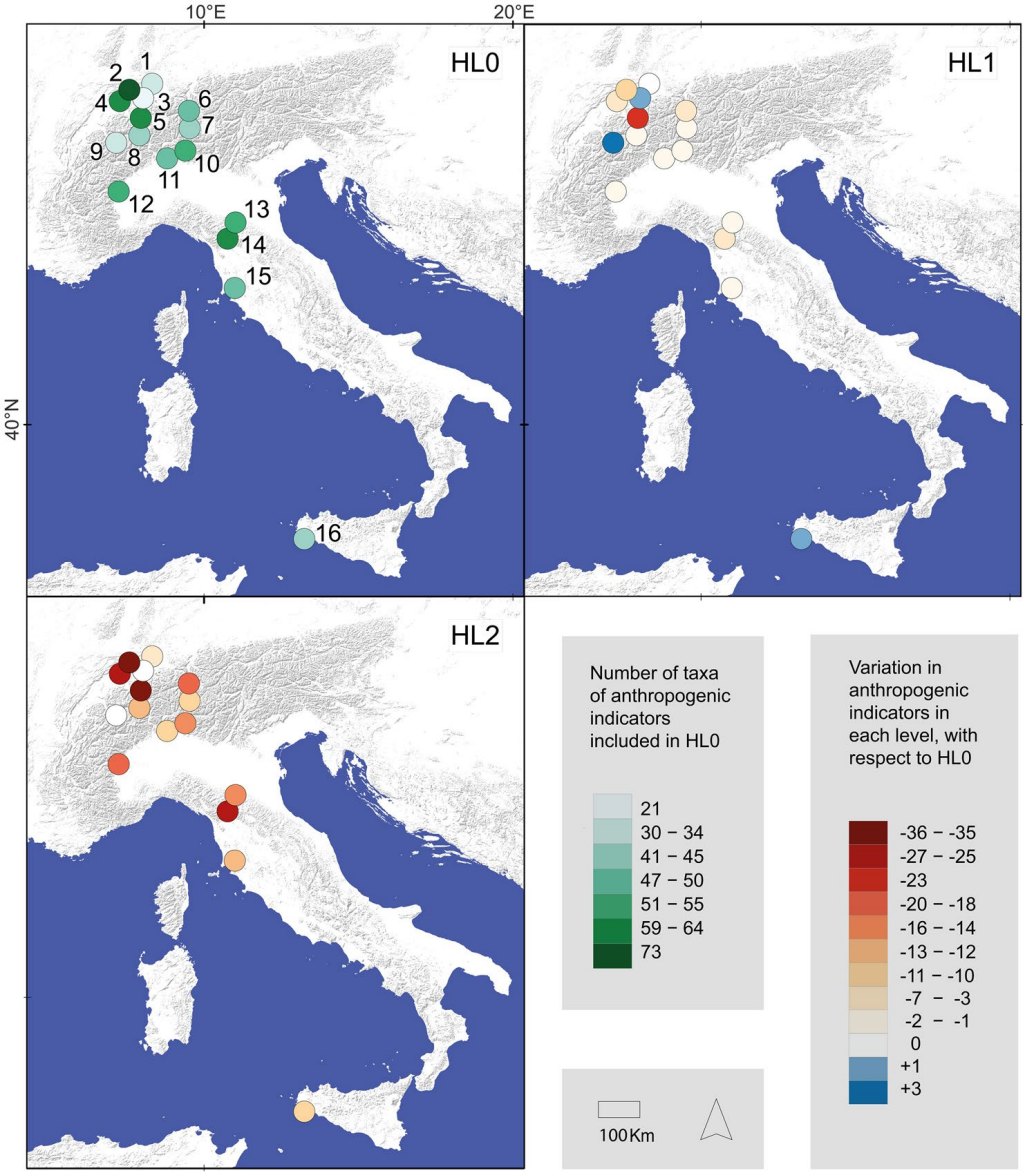
Location of the study sites and number of anthropogenic pollen types included in the hierarchical levels after harmonization (HL0-HL2) for each study site. 1—Egelsee (Menzingen), 2—Burgäschisee, 3—Soppensee, 4—Moossee, 5—Bachalpsee; 6—Lej da San Murezzan, 7—Lej da Champfèr, 8—Lengi Egga, 9—Gouillé Rion, 10—Lago di Origlio, 11—Lago di Muzzano, 12—Lago Piccolo di Avigliana, 13—Pavullo nel Frignano, 14—Lago del Greppo, 15—Lago dell'Accesa, 16—Gorgo Basso

We selected sixteen postglacial palaeoecological sequences along a latitudinal gradient encompassing from northern Switzerland to southern Italy (Sicily; Fig. 1), located at various altitudes (Table 2). These sequences were produced at the Palaeoecology Section of the University of Bern, thus allowing for consistent and high taxonomic resolution. The pollen datasets were obtained from the Alpine Paleoecological Database (ALPADABA) via Neotoma (Williams et al. 2018).

**Table 2:** Main features of the palynological records considered in this study

Site	Lat (°N), Long (°E)	Elevation (m a.s.l.)	Area (ha)	Age range (cal yr bp)	MAT (°C) (Fick and Hijmans 2017)	Reference
1. Egelsee (Menzingen)	47.183480, 8.582379	770	1.2	50–16,200	8.7	Wehrli et al. (2007)
2. Burgäschisee	47.148056, 7.658333	465	21	− 50–18,700	8.9	Rey et al. (2019a, b)
3. Soppensee	47.090421, 8.080115	596	22.7	− 50–14,200	8.6	Hajdas and Michczynski (2010; Lotter (1999)
4. Moossee	47.021944, 7.480278	521	31	3,850–7,100	9	Rey et al. (2019a, b)
5. Bachalpsee	46.670356, 8.023247	2,265	8	− 50–12,900	0.2	Lotter et al. (2006); van der Knaap et al. (2000)
6. Lej da San Murezzan	46.495168, 9.845067	1,768	78	− 50–11,900	1.9	Gobet et al. (2003, 2005); Henne et al. (2011)
7. Lej da Champfèr	46.471268, 9.807297	1,791	50	− 50–11,850	1.8	Gobet et al. (2003, 2005)
8. Lengi Egga	46.396840, 7.980020	2,557	2.89	10–12,600	− 0.6	Tinner and Theurillat (2003)
9. Gouillé Rion	46.157222, 7.362778	2,343	0.16	− 50–11,950	1.0	Tinner et al. (1996)
10. Lago di Origlio	46.060435, 8.942306	416	8	− 50–18,900	10.6	Tinner et al. (1999)
11. Lago di Muzzano	45.996621, 8.928177	337	22	− 50–15,150	11.5	Gobet et al. (2000); Tinner et al. (1999)
12. Lago Piccolo di Avigliana	45.050000, 7.383334	356	60	320–19,350	11.8	Vescovi et al. (2007)
13. Pavullo nel Frignano	44.318335 10.837500	675	ca. 20	100–16,300	12.6	Vescovi et al. (2010b)
14. Lago del Greppo	44.119722, 10.683055	1,442	0.018	− 50–14,950	6.7	Vescovi et al. (2010a)
15. Lago dell'Accesa	43.059388, 10.898260	157	16	50–11,600	14.2	Colombaroli et al. (2008); Finsinger et al. (2010)
16. Gorgo Basso	37.609174, 12.654939	6	3	− 55–10,200	18.1	Tinner et al. (2009)

### Taxonomic resolution of human pollen indicators

A bibliographic search yielded a list of plants which are associated with farming: primary indicators (plants cultivated in fields, orchards and gardens) and secondary indicators (weeds) classified into adventives and apophytes (ESM 1; Behre 1990; di Castri et al. 1990; Lang 1994; Chytrý et al. 2008; Sõukand and Kalle 2015). Adventives are non-native plants that were introduced with the agriculture (or locally very rarely before agriculture), whereas apophytes are native plants that were favored by human land use (Behre 1981; Lang 1994). This concept also applies to the crops. Some crops are native to Europe, while others were introduced. Here we apply the concept of adventive plants only to the weeds, but pay particular attention to whether a crop was native or not to the area. Indeed, most European crops originate from Southern Europe (e.g. fruit trees or lianas such as *Castanea sativa, Juglans regia*, *Olea europaea* or *Vitis vinifera*). By assembling this species list, we aimed to refine the anthropogenic pollen types traditionally used at lower taxonomic resolution such as at genus or family level (Table 1; Behre 1981). From the pollen types in our datasets, we selected all those that corresponded to anthropogenic pollen indicators at different taxonomic resolutions (see Table 1), resulting in a list of potential pollen indicators (ESM 2).

**Fig. 2:**
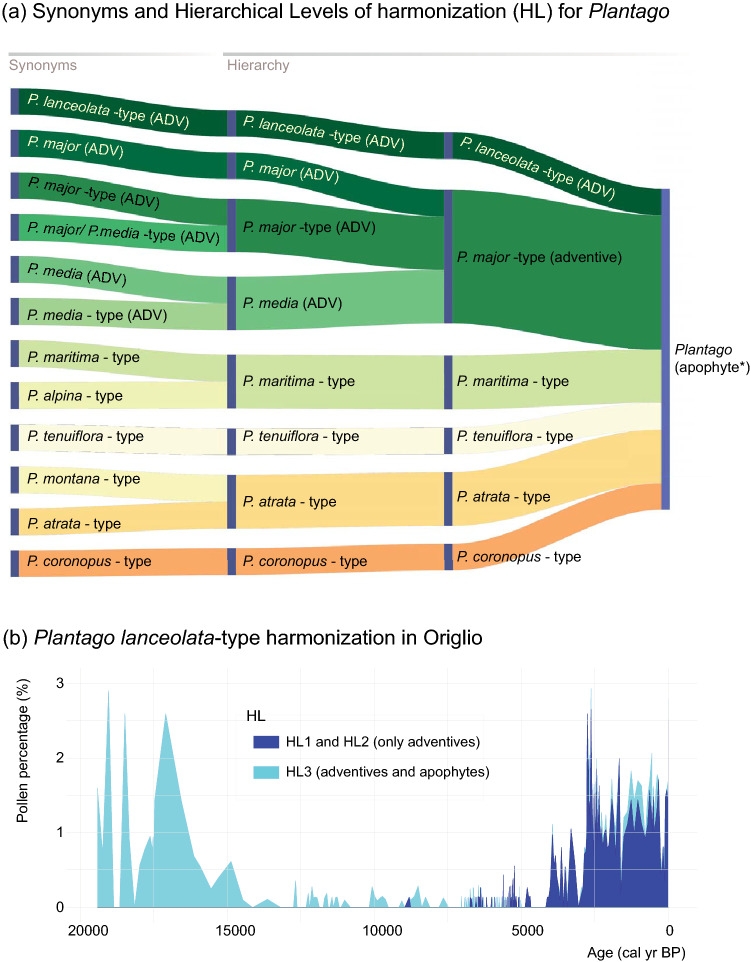
**a** Pollen type synonyms (Var.) and hierarchical levels of taxonomic resolution (HL0-HL2) and their relationship as in Giesecke et al. (2019). Example for *Plantago* and its constituent pollen types found in our study sites. Discontinuous line denotes exclusion of the pollen type as a human indicator in that harmonization level. (*) *P. lanceolata*-type and *P. major/media*-type are considered as adventives (ADV), but move to the apophyte category (APO) when they are merged with the other pollen types (e.g. *P. maritima*-type, *P. atrata*-type) into *Plantago*-type. **b** Results of the harmonization to different HL levels for *P. lanceolata*-type at the site Origlio (Tinner et al. 1999). Here, *P. lanceolata*-type is an (independent) adventive with high human-impact value at the levels HL0 and HL1 but moves together with other pollen types (e.g. *P. alpina*-type, *P. montana*-type) into the far less diagnostic apophyte category *Plantago*-type at the level HL2 (see high natural abundances at HL2 before agriculture started at ca. 7,500 cal bp). All levels used and included in the analysis are found in ESM 2

The nomenclature of the potential pollen indicators was first standardized according to the accepted pollen type names of the European Pollen Database (Giesecke et al. 2019). The starting point is a list of all the pollen type names accepted in the EPD (once synonymies were solved), assigned by Giesecke et al. (2019) to the base hierarchical level HL0. This level represents the highest taxonomic resolution currently attainable using sophisticated light microscopy (e.g. 1,000 × magnification, phase contrast), generally referring to genera, groups of closely related genera, families or, more rarely, species or groups of species. Based on HL0, Giesecke et al. (2019) constructed two hierarchical levels (HL1, HL2) that rely on pollen-morphological features and possess a decreasing taxonomic resolution. The first level (HL1) provides a list of morphologically similar pollen types identified when a good reference collection is available. The second hierarchy level (HL2) combines types with distinctive, mainly readily identifiable features often corresponding to groups of related plant genera or to families. HL2 combines pollen types that can be easily identified but are differently used in palaeoecology. For instance, it is widely used for *Plantago* but rarely for Poaceae-type, which includes also Cerealia-type at HL2. We selected the anthropogenic pollen indicators to be included in each simulated dataset according to the respective taxonomic level of harmonization HL0 to HL2 (e.g. for *Plantago* see Fig. 2a). At each taxonomic hierarchical level, pollen types deriving from plants that are not cultivated or not considered as adventives or apophytes were disregarded because of their diagnostic irrelevance for land use detection (ESM 2). The harmonization was performed using the packages “*tidyverse*” version 1.3.0 (Wickham et al. 2019a) and “*readxl*” version 1.3.1 (Wickham et al. 2019b) running in the R environment (R Core Team 2018), and resulted in two simulated datasets for each study site. Figure 1 reports the number of taxa and their variations in each hierarchical level with respect to the base level (HL0) for each considered site and taxonomic hierarchical level.

Within each harmonized sequence, we assigned a category of indicative capacity according to the synanthropic status found in literature (ESM 1). Introduced (or partly introduced) primary indicators have the highest indicative capacity, followed by plants considered to be adventives, and finally, the apophytes (Behre 1981, 1990; Lang 1994, ESM 2). When decreasing taxonomic precision changed the constitutive taxa of a given pollen type, we also lowered its indicative capacity. For instance, *Plantago lanceolata*-type and *P. major*-type are considered adventives at the highest taxonomic resolution levels HL0 and HL1. However, at level HL2 these pollen types are merged with other *Plantago* pollen types with no human indicator capacity into *Plantago*, which is thus regarded as an apophyte (Fig. 2).

### Statistical analysis

#### Pollen percentage calculation and Wilcoxon test

**Fig. 3:**
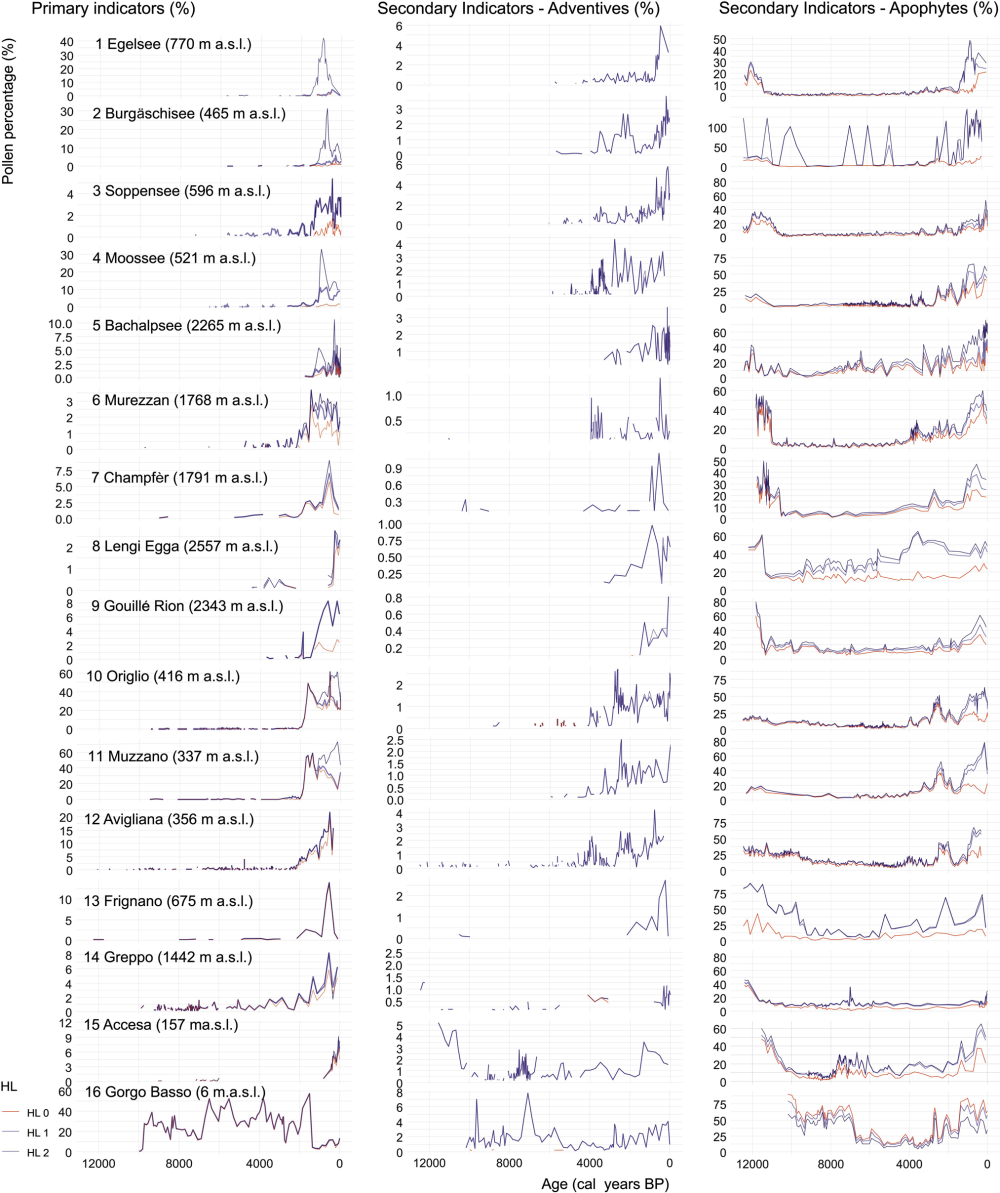
Anthropogenic pollen curves (as percentages of the reference sum including terrestrial pollen and fern spores) in each study site at the different hierarchical levels of harmonization (HL0 to HL2, with HL0 representing the highest and HL2 the the lowest level of taxonomic resolution) for each category of indicative capacities: Primary Indicators refer to crops and Secondary Indicators to weeds; adventives are plants that were involuntarily introduced alongside farming; apophytes are synanthropic species that are native in origin. In parenthesis, the altitude of the site; note the different scales in the y-axes; all graphics are constrained to the last 12,000 calendar years

Pollen percentages were calculated with respect to a reference sum that included the pollen of trees, shrubs and upland herbs, and the spores of terrestrial ferns. We included fern spores in the reference sum because *Pteridium aquilinum* and *Polypodium* are both considered secondary human indicators (Table 1; Behre 1981). The sum of pollen percentages of human indicators at each hierarchical level and each category of indicative capacity was plotted against time (Fig. 3).

**Fig. 4:**
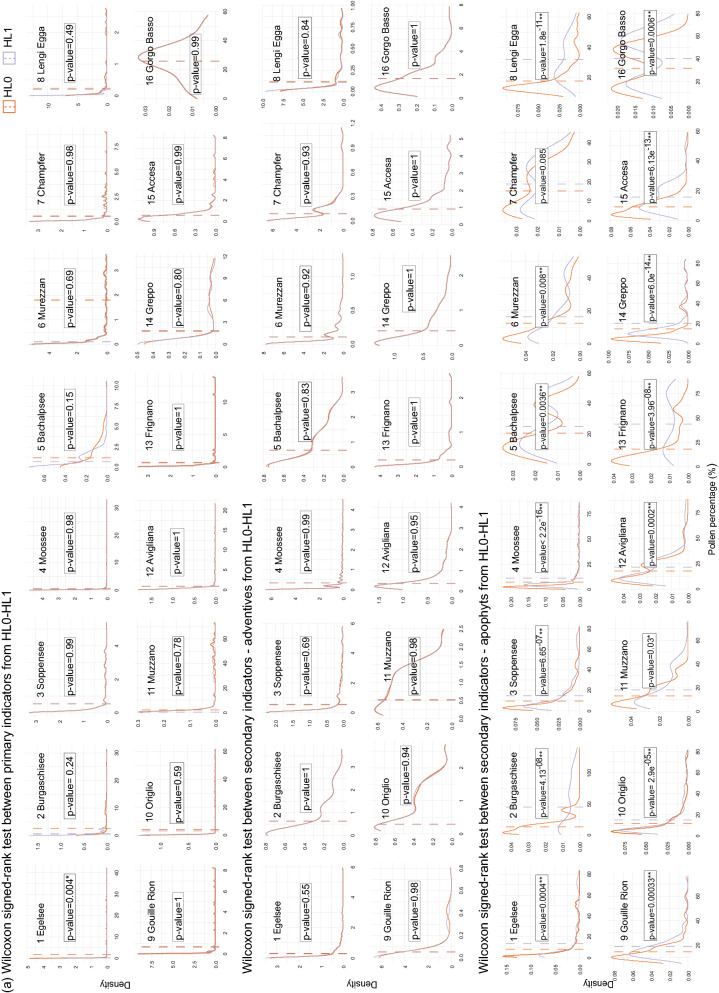

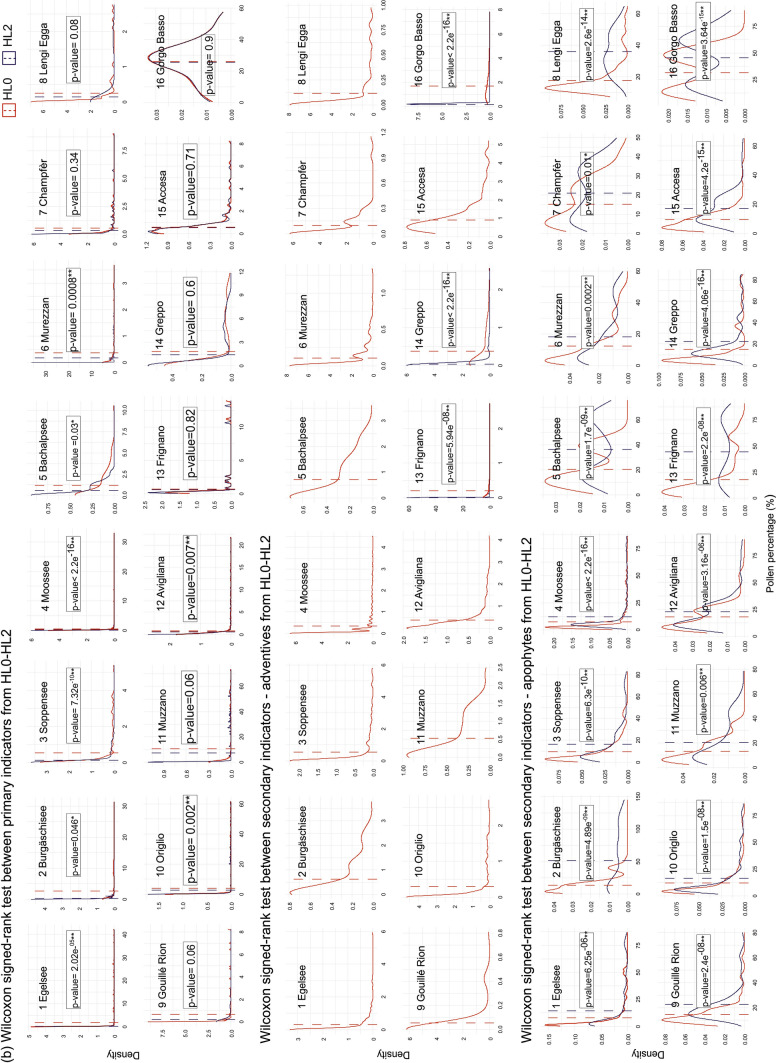
Kernel density (Sheather and Jones 1991) of anthropogenic pollen percentages in each study site, comparing the base level (HL0) sequences and the others: **a** HL0-HL1; and **b** HL0-HL2. The mean is shown for each curve. Each graph shows the statistical significance (p value) between the two hierarchical levels using Wilcoxon signed-rank paired test (Wilcoxon 1945). Statistically significant differences at p < 0.05 and p < 0.01 are denoted with * and **, respectively. Graphs not showing a p value correspond to the ones with no data at HL2

Our null hypothesis (H_0_) is that the abundance of human pollen indicators does not depend on the taxonomic hierarchical level (HL0 to HL2), meaning that decreasing taxonomic resolution does not bring any information loss. To test this hypothesis, in a first step we plotted the Kernel density (Sheather and Jones 1991) of anthropogenic pollen percentages between the base resolution level HL0 and the subsequently lower resolution levels HL1 and HL2 for each category of indicative capacity, i.e. primary indicators, adventives and apophytes. In a second step, we ran Wilcoxon signed-rank paired tests (Wilcoxon 1945) between them (Fig. 4). For these analyses, we used the R package “*plyr*” version 1.8.4 (Wickham 2011).

#### ‘Distantia’ analyses

To quantify the influence of the decrease in the taxonomic resolution of human indicators on their indicative value, we calculated the dissimilarities between the assemblages of anthropogenic pollen types (pollen percentages with respect to the reference sum defined above) among HL sequences with the R package “*distantia*” version 1.0.2 (Benito and Birks 2020). This package computes the dissimilarity measure Ψ or psi (Gordon and Birks 1974) between two multivariate ecological time‐series (METS). Given two METS (A and B, with lengths m and n), the dissimilarity measure Ψ is calculated as the sum of distances between their respective samples (AB_between_), normalized by the sum of distances between the consecutive samples within each sequence (AB_within_), plus 1 (in the case of including diagonals in the calculation to find the least-cost path):

$$ \psi = \left( {\left( {{\text{AB}}_{{{\text{between}}}} - {\text{AB}}_{{{\text{within}}}} } \right)/{\text{AB}}_{{{\text{within}}}} } \right) + 1. $$

The computation of the distance between their respective samples (AB_between_) and between consecutive samples (AB_within_) was made using the Euclidean method. In a first step, we quantified the influence of taxonomic resolution of anthropogenic pollen types on the dissimilarity among the three sequences HL0–HL2 (Table 3). This first dissimilarity analysis was based on the variables “anthropogenic pollen types” and the sequences were ordered along “depth/age”.

**Table 3:** Dissimilarity measure Ψ or psi values between pairs of HL sequences (Benito and Birks 2020)

Site	HL0-HL1	HL0-HL2	HL1-HL2
1. Egelsee (Menzingen)	1.212	1.244	0.198
2. Burgäschisee	2.296	2.024	0.220
3. Soppensee	1.443	1.531	0.368
4. Moossee	1.269	1.289	0.209
5. Bachalpsee	0.405	0.885	0.744
6. Lej da San Murezzan	0.865	0.932	0.356
7. Lej da Champfèr	0.514	0.624	0.290
8. Lengi Egga	2.780	2.826	0.439
9. Gouillé Rion	0.930	1.063	0.499
10. Lago di Origlio	0.563	0.617	0.258
11. Lago di Muzzano	0.444	0.552	0.330
12. Lago Piccolo di Avigliana	0.872	0.912	0.229
13. Pavullo nel Frignano	2.021	2.019	0.067
14. Lago del Greppo	1.137	1.367	0.818
15. Lago dell'Accesa	1.584	1.712	1.846
16. Gorgo Basso	0.493	0.553	0.278

This analysis quantifies the influence of the taxonomic resolution of anthropogenic pollen types on the dissimilarity among the three sequences HL0 to HL2. For each site throughout the entire time sequence, higher values indicate higher dissimilarities

**Fig. 5:**
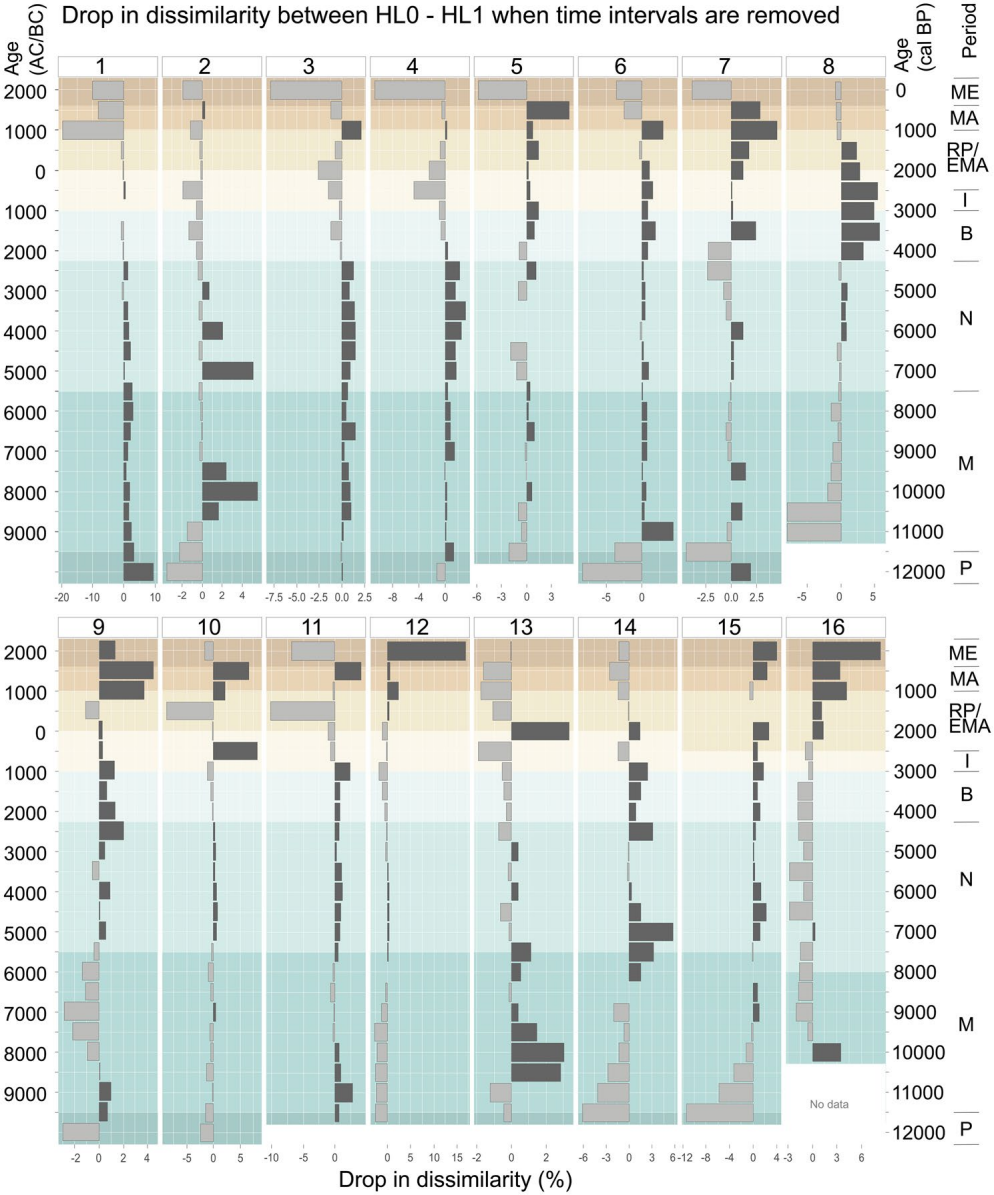
Drop in dissimilarity measure Ψ or psi values, in percentages, when the given time interval is removed from the dissimilarity analysis between HL0-HL1 hierarchical levels. This analysis was done with the purpose of quantifying the influence of certain periods on the dissimilarity between HL0 and HL1 sequences. Time intervals are 500 years. Large positive numbers (dark grey) indicate increase in similarity when the given time interval is removed, disclosing the relevance of the time interval for dissimilarity between both hierarchical levels (HL0-HL1). Negative values (light grey) indicate an increase in dissimilarity between the hierarchical levels when the time interval is dropped. The numbers in the graphics follow the nomenclature of sites in Fig. 1 and Table 2. Archaeological and historical periods: P—Palaeolithic, M—Mesolithic, N—Neolithic, B—Bronze Age, I—Iron Age, RP—Roman Imperial Period, EMA—Early Middle Ages, MA—Middle Ages, ME—Modern Era. Note the different scales in the X-axes. All graphics are constrained to the last 12,000 cal years

In a second step, we ordered the sequences along pollen-types and ran an analysis based on the variable “time intervals” with the goal of identifying the periods and taxa responsible for the dissimilarity. This second dissimilarity analysis was made to disclose the relevance of removed time intervals on the overall dissimilarity between hierarchical levels HL0-HL1 at a specific site. This analysis focused on HL0-HL1 because the lowest level HL2 is only moderately used by palynologists (e.g. for *Plantago*). The values of the variables “time intervals” were calculated by grouping pollen percentages into 500-year bins (average considering only non-zero values). The results are plotted as percentage drop in psi values for the remaining time variables, graphically allocated to the removed period (Fig. 5). Once a high drop in psi values was detected for a specific time interval with the analysis, we could identify in the pollen diagrams which were the taxa responsible for that change.

#### Change point analyses

To investigate long-term variations in the cumulative curve of all anthropogenic pollen taxa between the HL sequences, we used the “*changepoint*” v1.1.5 package of R (Killick and Eckley 2014; R Core Team 2018) to determine the time of significant human (and environmental) changes by each taxonomic hierarchical level (Killick et al. 2012). The change point analysis considers the variations between HL0, HL1 and HL2 concerning the mean and variance of the summary anthropogenic pollen records. We defined a maximum number of four change points to search for and constrained the datasets to the last 12,000 years to enhance inter-site comparison (ESM 3), applying the binary segmentation method (Scott and Knott 1974). This method is an iterative search method that tests if a change point at position τ exists that separates an ordered sequence of data (y1: n = (y1, …,yn)) into two segments (y1:τ = (y1, …,yτ), yτ + 1:n = (yτ + 1, …,yn)) at each iteration. A change point is taken in a given interaction when a cost function applied to the entire sequence (Ω1: n) is larger than the sum of the cost functions applied separately to the two segments plus a penalty β to guard against overfitting (Ω1: τ + Ωτ + 1: n + β), until no change points that meet this condition are detected (Killick et al. 2012). Change point analysis is a method increasingly used in palaeoecology to determine major shifts in time-series (e.g. Giesecke et al. 2014; Rius et al. 2014; Finsinger et al. 2018).

## Results

### Effect of HL harmonization in each site and pollen type

The harmonization process resulted in a variation of the number of indicative taxa among all study sites (Fig. 1). At the base level HL0, a slight latitudinal increase of anthropogenic taxa towards the southern sites was found, although this trend is blurred by the specific characteristics of each site (mainly altitude) and the resolution achieved in the dataset. Taxonomic harmonization at the three hierarchical levels shows a significant reduction in the total number of potential anthropogenic pollen types from HL0 to HL2 (179 in HL0, 121 in HL1, and 49 in HL2 (ESM 2). Similarly, the overall number (all sites) of pollen types with indicative value for anthropogenic impact used in the simulated datasets dropped from HL0 to HL2 (80 in HL0, 63 in HL1, and 42 in HL2). The total number of anthropogenic indicator taxa remains mostly constant at site level between HL0 and HL1 but shows a dramatic drop in HL2 at most sites (Fig. 1).

**Fig. 6:**
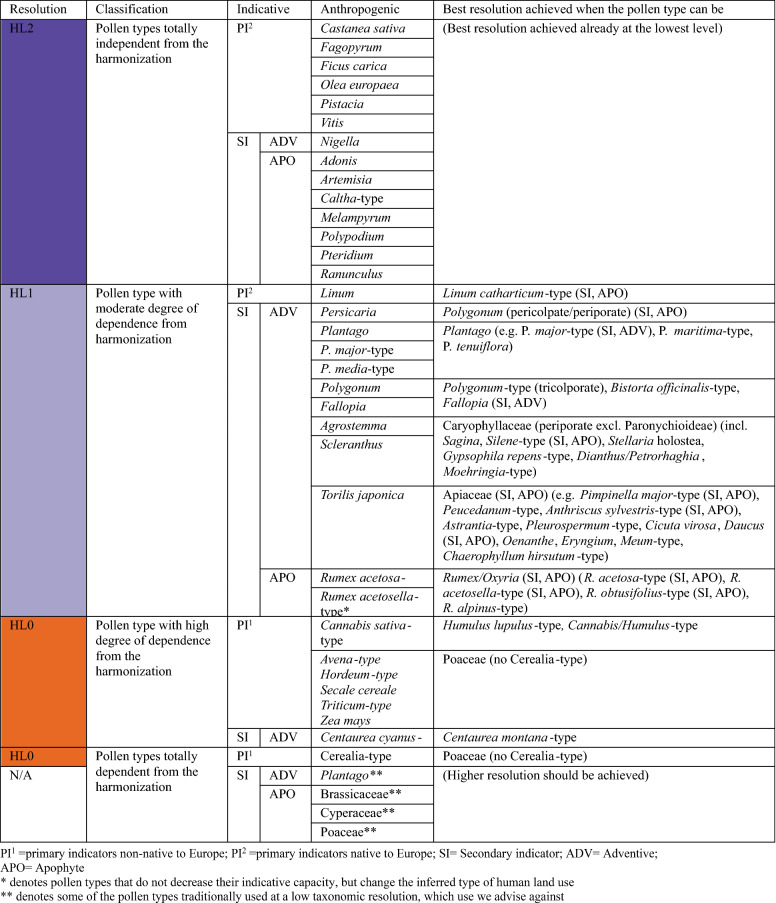
Classification of main anthropogenic pollen types by the degree of effect that the taxonomic harmonization had on its indicative capacity. Pollen types totally independent from harmonization did not change their anthropogenic indicative capacity at any level (HL0-HL1-HL2). For each anthropogenic indicator, we indicate the highest resolution level, so that it is not confused with other pollen types with different indicative capacity. For a detailed explanation of the taxonomic harmonization list, see ESM 2

A first group of anthropogenic pollen types with indicator capacity was totally independent from the harmonization and consisted of easily identifiable taxa (even at the lowest HL2) such as *Artemisia, Adonis, Caltha*-type*, Ranunculus arvensis*-type (apophytes), *Nigella* (adventive), *Fagopyrum, Olea europaea, Castanea sativa, Ficus carica, Vitis* and *Pistacia* (introduced or native primary indicators). On the other hand, the indicative value of a numerous array of pollen types clearly depends on the taxonomic resolution attained, although the magnitude of this effect is variable. At the lowest degree of dependence, the following group of taxa bear constant indicative capacity through HL0 and HL1 levels since their precise identification (at species or genus level) is often achieved, although a decrease in indicative power at HL2 must be noted. These taxa are the primary indicator *Linum usitatissimum* and most of the secondary adventives (*Agrostemma githago*, *Fallopia*, *Persicaria maculosa*-type, *Plantago lanceolata*-type*, P. major*-type, *P. media*-type, *Polygonum aviculare*-type, *Scleranthus*, *Torilis japonica*); in this group we also have important taxa such as *Rumex acetosa*-type and *R. acetosella-*type, which do not change their indicative value with varying taxonomical resolution (they are both apophytes), but do change the inferred type of human land use (*R. acetosa*-type is a herb indicator of grasslands and *R. acetosella-*type is indicative of ruderal vegetation). With a moderate degree of dependence from harmonization, we have the primary indicator *Cannabis sativa*-type and adventive *Centaurea cyanus*-type*.* In contrast, the group most strongly affected by the harmonization in terms of indicator value included principally human pollen types originally defined at low taxonomic resolution such as Cerealia-type, Poaceae, Brassicaceae, Cyperaceae and *Plantago* (Fig. 6).

### Effect of the harmonization on the cumulative curves of anthropogenic indicators

The cumulative curves of anthropogenic pollen according to their category of indicative capacity showed differences resulting from the harmonization process (Fig. 3). However, when comparing HL0 and HL1 only the curves of apophytes were significantly different, unlike those of all the primary indicators and the adventives (Fig. 4). The influence of the harmonization was stronger when considering HL0 and HL2, particularly on the abundances of secondary (both adventives and apophytes) and primary human indicator types, mostly in the northern sites (Fig. 4). However, at most sites adventives were lacking at HL2 because they were re-assigned to the apophyte category (Figs. 2, 3 and 4).

The largest dissimilarity values (Ψ or psi values) in the assemblages of anthropogenic pollen indicators were found between the base level HL0 and the lowest level of resolution HL2, except at Burgäschisee (site #2), which was originally produced at particularly high taxonomic resolution (73 anthropogenic taxa in HL0 compared to 63 in HL1), at Pavullo nel Frignano (site #13), where the outstanding abundance of Cyperaceae pollen in HL1 enhances the HL0-HL1 dissimilarity, and at Accesa (site #15), where the change in the number of taxa is even more drastic between HL1 (46 taxa) and HL2 (34 taxa; Table 3).

### Time variations of human pollen types due to HL harmonization

Estimation of the land use impact tends to be higher at lower levels of resolution. When the dissimilarity was estimated on the basis of time intervals and the contribution of each time interval to dissimilarity was calculated, the highest levels of dissimilarity between the HL0 and HL1 sequences were found mainly during the Mesolithic and Neolithic (Fig. 5). At Egelsee (site #1), Soppensee (site #3), and Muzzano (site #11), while the dissimilarity is slightly higher in periods such as the Paleolithic when land use is not detectable or minimal. In other cases, and especially at high altitude sites, the highest dissimilarity occurs after the Bronze Age due to Cyperaceae, Poaceae, Brassicaceae and Ericaceae (tetrads) included in HL1, as at Murezzan (site #6 at 1,768 m a.s.l.), Champfer (site #7 at 1,791 m a.s.l.) and Lengi Egga (site #8 at 2,557 m.a.s.l.). The dissimilarity at Egelsee (site #1) is also very high (up to 10%) in the Modern Era. Within the Mediterranean sites, at Gorgo Basso (site #16) the dissimilarity is concentrated at the beginning of the record during the Mesolithic [due to Poaceae, Ericaceae (tetrads) and Brassicaceae in the older samples] and after the early Middle Ages, with up to 4% of dissimilarity change mainly due to Apiaceae. The change point analysis of the anthropogenic pollen indicators (ESM 3) identified mainly the environmental change at the onset of the Holocene, the human-induced vegetation change around the Neolithic, and the land use intensification around the Bronze Age. Overall, the detected changes coincide in time, with some rare exceptions. For instance, at Lengi Egga (site #8 at 2,557 m a.s.l.) the highest resolution HL0 displays a later change point in land-use intensification with respect to HL1 and HL2. At other sites, HL0 presented earlier change points than HL1 and HL2 at the onset of the Holocene, as at Lago Piccolo di Avigliana (site #12) and Lago del Greppo (site #14).

## Discussion

Pollen-inferred reconstructions of human activity rely on the relationship between agricultural crops or weeds and their imprint in pollen assemblages (Behre 1981). Previous studies showed that high taxonomic resolution is important for the reconstruction of human impact from pollen records (Tinner et al. 1996; Seppä and Bennett 2003). Specifically, an accurate and precise pollen taxonomy is key to correctly identifying crops or weeds associated with land use and in defining diagnostic human-impact indices (Giesecke et al. 2014). Attaining the high taxonomic resolution needed may be time consuming and require a significant analytical effort, including consulting reference slides and using microscopes equipped with 1,000 × magnification (using immersion oil) and phase contrast (Tinner et al. 2007; Beug 2004; Rey et al. 2017). However, such additional work is worthwhile when aiming at detailed reconstructions of arable farming activities in prehistoric times, as the determination of different pollen types of cereals (e.g. *Avena*-type, *Hordeum*-type, *Triticum*-type) may deliver relevant information in this regard (Beug 2004; Rey et al. 2017). Likewise, it becomes essential to understand the possible effects of taxonomical losses related to the harmonization practices inherent in big data analysis, the use of diversity at higher taxonomic ranks (e.g. genus, family) as a surrogate for species diversity (Bevilacqua et al. 2012), as well as the use of functional types for compensating for existing taxonomic imprecisions (Gajewski 2008), particularly considering the prominence that these topics are reaching in recent times.

Here, we show that decreasing taxonomic resolution has noticeable effects when moving from highest to high (i.e. HL0–HL1) and from highest to intermediate (HL0–HL2) levels (Table 3, Figs. 3 and 4). Further, our results show that aiming at the highest possible taxonomic resolution level is important in distinguishing between adventive and apophyte taxa, i.e. in separating introduced crops and weeds from native plants. This distinction is important for disentangling the role of human impact from other natural drivers of vegetation change like climatic variability, fire disturbance or herbivory. Indeed, if all human indicators are put together at sites originally produced with high taxonomical resolution, the dissimilarity values are already remarkable between the base level HL0 and the immediately lower harmonization level HL1 (Table 3).

As agricultural crops are the foundation of farming economies, primary human indicators have the highest indicative value (Behre 1981). Caution is however needed in the interpretation of primary indicators that are native to an area (e.g. *Olea europaea* and *Vitis vinifera* in southern Europe). Here we show that primary indicators are mostly well recognized at the high levels HL0 and HL1 but might be overlooked at lower taxonomic resolution (HL2; see ESM 3). The highest loss in the indicative capacity of anthropogenic pollen indicators may occur when the pollen of primary indicators such as cereals cannot be distinguished from the pollen of wild grasses. In regard to the secondary indicators, adventives are particularly important because these taxa were introduced with agriculture. These plants therefore depend on human activities to thrive and are not naturally present in the vegetation, which makes them particularly well suited as indicators of human activity (Behre 1990; Lang 1994; Tinner et al. 2007), in contrast to apophytes. Remarkably, our results also show that the family or sub-family level may lead to confusion of natural and anthropogenic processes in human impact and land use reconstructions, so they should be avoided in such assessments. Low taxonomic resolution actually introduces an overrepresentation and thus an overestimation of naturally occurring apophytes, consequently reducing the diagnostic value of summary pollen curves of human impact. Such palynological considerations are in agreement with previous studies showing that species level is the most accurate taxonomic rank for bioindication analysis (Nahmani et al. 2006), even if this is seldom reached in reconstructions of past human impact.

The beneficial effect of enhanced taxonomic resolution can be illustrated with *Rumex* and *Oxyria*. Most palynologists do not distinguish the pollen of *Rumex* and *Oxyria*, considering all these grains as *Rumex*-type. This imprecise determination, however, may pose a relevant issue in arctic and alpine landscapes, where the generalised use of *Oxyria* or *Oxyria*-type (because *Oxyria* is a common plant there) may mask the presence/abundance of *Rumex acetosella*-type and *R. acetosa*-type and thus make the identification of human impact difficult. Furthermore, *R. acetosella*-type is considered an apophyte indicative of ruderal vegetation and arable farming and thus has more indicative value than *R. acetosa*-type, a grassland indicator (Behre 1981). Similarly, *Plantago*-type has adventive constituents (e.g. *P. lanceolata*-type) strongly related to land use but also apophytes that occur in natural environments (e.g. *P. alpina*-type, *P. montana*-type; Fig. 2). The determination of *Plantago* pollen at genus level is quite common in practice (Mercuri et al. 2013a) although it may hamper or even prevent the recognition of human impact. Our study also calls for attention to common and usually abundant plant taxa that have so far experienced little progress in their pollen taxonomy such as Poaceae or Cyperaceae. However, some promising technical innovation such as confocal microscopy might help in the future (Seppä and Bennett 2003). Interestingly, *Eriophorum* and *Carex* pollen (both apophytic Cyperaceae) could be amalgamated into *Dulichium*-type (Faegri and Iversen 1975), which might allow tentative reconstructions of land-use regimes (Fjordheim et al. 2018) in the absence of other more specific human indicators. Dissimilarities derived from taxonomic precision were very high (up to 10%) during the Modern Period (after ad 1600; Fig. 5). However, even if apparently minor, low taxonomic precision in the identification of anthropogenic pollen indicators can lead to overestimate human impact when investigating prehistoric periods such as the Mesolithic-Neolithic transition (Fig. 5). Given that in this initial stage crops and weeds were still rare and thus their pollen signature very weak, an accurate application of the human indicator method is crucial (Fig. 5; ESM 3). For instance, in Sicily, primary and secondary indicators of human activity were found during the Neolithic (after 8,000 bp, Fig. 3). Nevertheless, with the exception of *Ficus*, the evidence was ambiguous (Tinner et al. 2009), since plants producing the other anthropogenic pollen types (including Cerealia-type) were present in the Mediterranean natural vegetation prior to the onset of Neolithization ca. 8,000 years ago (Calò et al. 2012). Combining unambiguous and ambiguous pollen indicators of human activities (here the primary indicators *Ficus* and Cerealia-type) may contribute to solving this issue by reducing the probability of wrong attribution to human impact. Woody taxa such as *Juglans regia, Vitis vinifera* or *Olea europaea* are important primary indicators in southern Europe. The identification of these pollen indicators is robust and unequivocal, but if imprecise due to bad preservation they are often taxonomically downscaled. For example, Weiberg et al. (2019) grouped Oleaceae with *Olea* and considered it as a primary indicator*,* whereas other genera of Oleaceae such as *Fraxinus*, *Phillyrea* or *Jasminum* stayed separate. Similarly, the same authors grouped Juglandinae with *Juglans* (to account for sporadic occurrences of *Carya* and *Pterocarya*). Importantly, the use of these taxa as primary indicators needs a thorough control of whether they were part of the native vegetation in the study area prior to the shift to production economy during the Neolithic (e.g. *Olea europaea* is native in thermomediterranean vegetation of Europe; Langgut et al. 2019) or not. Indeed, in the past years, pollen of these fruit trees has been increasingly used to identify human impact in southern Europe (e.g. Mercuri et al. 2013b; Woodbridge et al. 2019). However, these pollen types cannot be separated from their wild European ancestors or relatives even at the highest level of taxonomic resolution.

The integration of plant macrofossil analysis in archaeological settings (e.g. Tserendorj et al. 2021) may overcome issues related to wild and cultivated varieties producing the same pollen type as it allows the identification of cultivars of these species (Terral et al. 2004) and is particularly well suited for exploration of the transition from wild to cultivated varieties (Valamoti et al. 2020). The introduction of fruit trees occurred usually significantly later than that of basic crops such as cereals. Usually, fruit trees boost the human impact curves since the Iron or Roman Age (Fig. 3), when they were introduced in non-Mediterranean areas (Conedera et al. 2004; Pollegioni et al. 2017). In contrast to *Vitis vinifera* and *Olea europaea*, which were widespread in southern Europe before the onset of farming, the natural rarity of *Castanea sativa* and *Juglans regia* (Krebs et al. 2019) make them good indicators of post-Neolithic human impact all over Europe.

Difficulties may arise when an indicator species is native to an area but adventive in another (Moore et al. 1991; Deza-Araujo et al. 2020), specifically in supra-regional syntheses. The different status of a pollen type in different regions implies that pollen-based reconstructions of human impact should always account for the specific local to regional conditions. Specifically, pollen types which are good proxies for anthropogenic disturbance in temperate or boreal Europe north of ca. 45°N are less reliable when applied in southern Europe, where many pollen types (e.g. *Ammi, Anemone, Apium*, *Bupleurum*, *Cardamine*, *Centaurea montana*-type, *Clematis*, *Delphinium, Filago*-type, *Gnaphalium*-type, *Jasione montana*, *Linum austriacum*-type, *Matthiola*, *Secale*, *Scabiosa*, *Silene*) derive from both anthropogenically introduced and natural species. As in the temperate and boreal areas of Europe, the ambiguity of pollen interpretation is highest when considering families with poorly differentiated pollen types such as Apiaceae, Brassicaceae, Caryophyllaceae and Poaceae, all very diversified in the Mediterranean region. Extreme cases are represented by Amaranthaceae, including many shrubs linked to salty and seasonally inundated clayey soils (e.g. *Suaeda*, *Halocnemum*, *Salsola*, *Salicornia* s.l., *Atriplex* s.l.) growing in the coastal marshes or colonizing the inland badlands. Here, the problems derived from palynological taxonomical imprecision may be mitigated by the inclusion of other palaeoecological proxies such as sedimentary ancient DNA and molecular biomarkers. These novel approaches are particularly useful when refined resolution is not possible through pollen analysis (Dubois and Jacob 2016). For instance, cannabinol (an organic molecule specific to hemp) allows the distinction of *Cannabis* from *Humulus* (Lavrieux et al. 2013) two taxa that in palynology have some taxonomic overlaps (Moore et al. 1991).

## Conclusions

The use of taxonomically poorly resolved anthropogenic indicators is a widespread practice in palaeoecology. Such taxonomic imprecision may bias the determination of human impact on past environments, given that natural species might be considered as anthropogenic and vice-versa. Moreover, the nature of land use activities might not be recognized correctly (e.g. different crops and weeds associated with arable and pastoral farming). In general, pollen datasets at low taxonomical resolution tend to overestimate land use and to produce false positives in detecting human presence during periods of very low or insignificant human impact. To overcome these difficulties, we provide a detailed list of key pollen types that require adequate taxonomic resolution for the identification of human impact (Fig. 6). Where an accurate pollen identification is not possible, questions related to human impact identification may remain open. To some extent, this is also true for the highest taxonomic resolution that can be reached today with palynology. Given that the diagnostic capacity depends on the identification of species or even varieties of plants, further studies refining the taxonomic level of important taxa are in any case needed. Substantial improvements may derive from taxonomic enhancements related to new techniques, such as biochemical analyses and sedimentary ancient DNA. Our study also has implications for multidisciplinary research in this topic, for instance we expect further progress from a better integration of palaeoecological, archaeobotanical, archaeological and historical approaches. Future multi-proxy studies focusing on increasing the taxonomic level of determination of crops and adventives may provide new insights on the land use history of Europe.

## Supplementary Information

Below is the link to the electronic supplementary material.

Supplementary file1 (DOCX 81 KB)

Supplementary file2 (DOCX 59 KB)

Supplementary file3 (DOCX 600 KB)
