# Supplementary material for: Influence of taxonomic resolution on the value of anthropogenic pollen indicators
Source: Veg Hist Archaeobot. 2021 May 11;31(1):67–84. doi: 10.1007/s00334-021-00838-x (PMC8738506; doi:10.1007/s00334-021-00838-x)
Supplement: Supplementary file 1 — Supplementary file1 (DOCX 81 KB) [file 334_2021_838_MOESM1_ESM.docx]

*Article title: “*Influence of taxonomic resolution on the value of anthropogenic pollen indicators”

*Journal name:* Vegetation History and Archaeobotany

*Author names:* Mara Deza-Araujo; César Morales-Molino; Marco Conedera; Gianni B. Pezzatti; Salvatore Pasta; Willy Tinner

*Affiliation and e-mail address of the corresponding author*: Insubric Ecosystems, Swiss Federal Institute for Forest, Snow and Landscape Research WSL, Cadenazzo, Switzerland and

Institute of Plant Sciences and Oeschger Centre for Climate Change Research, University of Bern, Bern, Switzerland - mara.deza@wsl.ch

**ESM 1** Plant species with anthropogenic indicative capacity in palaeoecological studies, according to their relative importance in archaeological/ historical periods. Evidence of selected species by macro-remains and pollen records in the different cultural epochs. After Lang (1994), Willerding (1986) and Knörzer (2007). (*) species that were not present in our study records. (**) plant taxa that belong to human indicators traditionally defined at lower taxonomic resolution (family, subfamily)

| Human indicator | Family | Origin | Neolithic | Bronze Age | Pre-Roman Iron Age | Roman Period | Middle Ages | Older modern Times | Younger modern Times (after 1800 AD) |  |
| --- | --- | --- | --- | --- | --- | --- | --- | --- | --- | --- |
| PRIMARY INDICATORS (Cultivated crops) | | | | | | | | | | |
| CARBOHYDRATE PLANTS | | | | | | | | | | |
| *Triticum aestivum/durum* | Poaceae** | W.As | ⃝ | ⃝ | ⃝ | ⃝ | ⃝ | ⃝ | ⃝ |  |
| *Triticum dicoccum* | Poaceae** | W.As | ⃝ | ⃝ | ⃝ | ⃝ | ⃝ | ⃝ | ⃝ |  |
| *Triticum monococcum* | Poaceae** | W.As | ⃝ | ⃝ | ⃝ | ⃝ | ⃝ | ⃝ | ⃝ |  |
| *Triticum spelta* | Poaceae** | W.As | ⃝ | ⃝ | ⃝ | ⃝ | ⃝ | ⃝ | ⃝ |  |
| *Hordeum vulgare* | Poaceae** | W.As | ⃝ | ⃝ | ⃝ | ⃝ | ⃝ | ⃝ | ⃝ |  |
| *Avena sativa* | Poaceae** | W.As |  | ⃝ | ⃝ | ⃝ | ⃝ | ⃝ | ⃝ |  |
| *Panicum miliaceum** | Poaceae** | C.As | ⃝ | ⃝ | ⃝ | ⃝ | ⃝ | ⃝ | ⃝ |  |
| *Setaria italica* | Poaceae** | As | ⃝ | ⃝ | ⃝ | ⃝ | ⃝ | ⃝ | ⃝ |  |
| *Secale cereale* | Poaceae** | W.As |  |  | ⃝ | ⃝ | ⃝ | ⃝ | ⃝ |  |
| *Zea mays* | Poaceae** | C.Am |  |  |  |  |  | ⃝ | ⃝ |  |
| *Fagopyrum esculentum* | Polygonaceae | E.As |  |  |  |  | ⃝ | ⃝ | ⃝ |  |
| *Solanum tuberosum** | Solanaceae | S.Am |  |  |  |  |  | ⃝ | ⃝ |  |
|  | | | | | | | | | | |
| PROTEIN PLANTS | | | | | | | | | | |
| *Pisum sativum* | Fabaceae | W.As | ⃝ | ⃝ | ⃝ | ⃝ | ⃝ | ⃝ | ⃝ |  |
| *Lens culinaris** | Fabaceae | W.As | ⃝ | ⃝ | ⃝ | ⃝ | ⃝ | ⃝ | ⃝ |  |
| *Vicia faba* | Fabaceae | W.As |  | ⃝ | ⃝ | ⃝ | ⃝ | ⃝ | ⃝ |  |
| *Phaseolus vulgaris** | Fabaceae | C.+S.Am |  |  |  |  |  | ⃝ | ⃝ |  |
| *Cicer arietinum* | Fabaceae | W.As | ⃝ | ⃝ | ⃝ | ⃝ | ⃝ | ⃝ | ⃝ |  |
|  | | | | | | | | | | |
| OIL AND FIBRE PLANTS | | | | | | | | | | |
| *Linum usitatissimum* | Linaceae | Eur,W.As | ⃝ | ⃝ | ⃝ | ⃝ | ⃝ | ⃝ | ⃝ |  |
| *Cannabis sativa* | Cannabaceae | C.As |  |  | ⃝ | ⃝ | ⃝ | ⃝ | ⃝ |  |
| *Papaver somniferum** | Papaveraceae | ? | ⃝ | ⃝ | ⃝ | ⃝ | ⃝ | ⃝ | ⃝ |  |
| *Camelina sativa** | Brassicaceae** | E.Med | ⃝ | ⃝ | ⃝ | ⃝ | ⃝ | ⃝ | ⃝ |  |
| *Brassica rapa** | Brassicaceae** | Eur | ⃝ | ⃝ | ⃝ | ⃝ | ⃝ | ⃝ | ⃝ |  |
|  | | | | | | | | | | |
| VEGETABLES | | | | | | | | | | |
| *Cucumis sativus** | Cucurbitaceae | S.As |  |  | ⃝ | ⃝ | ⃝ | ⃝ | ⃝ |  |
| *Cucumis melo** | Cucurbitaceae | S.As,Afr |  |  | ⃝ | ⃝ | ⃝ | ⃝ | ⃝ |  |
| *Lycopersicon esculentum** | Solanaceae | C.+S.Am |  |  |  |  |  | ⃝ | ⃝ |  |
| *Beta vulgaris** | Amaranthaceae* | Med,Atl |  |  | ⃝ | ⃝ | ⃝ | ⃝ | ⃝ |  |
|  | | | | | | | | | | |
| DYE PLANTS | | | | | | | | | | |
| *Reseda luteola** | Resedaceae | Med | ⃝ |  | ⃝ | ⃝ | ⃝ | ⃝ | ⃝ |  |
| *Isatis tinctoria** | Brassicaceae** | W.As |  |  | ⃝ | ⃝ | ⃝ | ⃝ |  |  |
| *Rubia tinctorum** | Rubiaceae | SW.As |  |  | ⃝ | ⃝ | ⃝ | ⃝ | ⃝ |  |
|  | | | | | | | | | | |
| FRUIT TREES AND NUTS | | | | | | | | | | |
| *Olea europaea* | Oleaceae | Med | ⃝ | ⃝ | ⃝ | ⃝ | ⃝ | ⃝ | ⃝ |  |
| *Juglans regia* | Juglandaceae | E.M,W.As |  | ⃝ | ⃝ | ⃝ | ⃝ | ⃝ | ⃝ |  |
| *Castanea sativa* | Fagaceae | W.As |  | ⃝ | ⃝ | ⃝ | ⃝ | ⃝ | ⃝ |  |
| *Ficus carica* | Moraceae | Med | ⃝ | ⃝ | ⃝ | ⃝ | ⃝ | ⃝ | ⃝ |  |
| *Citrus* div. spec.* | Rutaceae | SE.As |  |  | ⃝ | ⃝ | ⃝ | ⃝ | ⃝ |  |
| *Malus domestica** | Rosaceae | Eur | ⃝ | ⃝ | ⃝ | ⃝ | ⃝ | ⃝ | ⃝ |  |
| *Prunus avium** | Rosaceae | Eur | ⃝ |  | ⃝ | ⃝ | ⃝ | ⃝ | ⃝ |  |
| *Prunus domestica** | Rosaceae | W.As | ⃝ | ⃝ | ⃝ | ⃝ | ⃝ | ⃝ | ⃝ |  |
| *Vitis vinifera* | Vitaceae | Eur | ⃝ | ⃝ | ⃝ | ⃝ | ⃝ | ⃝ | ⃝ |  |

| APOPHYTES AND ARCHAEOPHYTES IN THE WEED AND RUDERAL VEGETATION OF CENTRAL EUROPE | | | | | | | |
| --- | --- | --- | --- | --- | --- | --- | --- |
|  | | APO | NL | BA | IA | RP | MA |
| *Aegopodium podagraria** | Apiaceae** | ? | *.* | *.* | O | O | O |
| *Carduus nutans** | Asteraceae | O | . | . | O | O | O |
| *Cirsium arvense** | Asteraceae | O | O | O | O | O | O |
| *Euphorbia cyparissias** | Euphorbiaceae | O | . | O | . | O | O |
| *Lamium album** | Lamiaceae | O | O | *.* | O | O | O |
| *Linaria vulgaris** | Plantaginaceae | O | O | . | O | O | O |
| *Malva sylvestris** | Malvaceae | O | O | O | O | O | O |
| *Mentha arvensis** | Lamiaceae | O | O | O | O | O | O |
| *Plantago lanceolata* | Plantaginaceae | ?/. | O | O | O | O | O |
| *Plantago major* | Plantaginaceae | O/. | O | O | O | O | O |
| *Plantago media* | Plantaginaceae | O/. | . | O | O | O | O |
| *Potentilla anserina** | Rosaceae | O | . | . | O | O | O |
| *Potentilla argentea** | Rosaceae | ? | O | *.* | *.* | O | O |
| *Ranunculus repens (other)** | Ranunculaceae** | O | O | O | O | O | O |
| *Rumex acetosella* | [Polygonaceae](https://en.wikipedia.org/wiki/Polygonaceae) | O | O | O | O | O | O |
| *Stellaria graminea (other)** | Caryophyllaceae** | O | O | O | O | O | O |
| *Urtica dioica* | Urticaceae | O | O | O | O | O | O |
| *Alliaria petiolata** | Brassicaceae** | *.* | O | . | . | O | O |
| *Arctium lappa ** | Asteraceae | *.* | O | O | . | O | O |
| *Arctium minus** | Asteraceae | *.* | O | O | O | O | O |
| *Artemisia vulgaris* | Asteraceae (Asteroideae)** | *.* | O | . | . | . | O |
| *Carduus crispus** | Asteraceae | *.* | O | . | O | O | O |
| *Chenopodium bonus-henricus** | Amaranthaceae** | *.* | O | O | O | O | O |
| *Cirsium vulgare** | Asteraceae | *.* | O | . | O | O | O |
| *Echium vulgare** | Boraginaceae | *.* | O | O | O | O | O |
| *Lapsana communis** | Asteraceae | *.* | O | O | O | O | O |
| *Malva moschata** | Malvaceae | *.* | O | . | . | . | . |
| *Melilotus officinalis** | Fabaceae | *.* | O | *.* | *.* | *.* | O |
| *Nepeta cataria** | Lamiaceae | *.* | O | . | O | O | O |
| *Picris hieracioides** | Asteraceae (Cichorioideae)** | *.* | O | O | O | O | O |
| *Saponaria officinalis* | Caryophyllaceae** | *.* | O | O | . | O | O |
| *Verbena officinalis** | Verbenaceae | *.* | O | O | O | O | O |
| *Veronica serpyllifolia** | Plantaginaceae | *.* | O | *.* | O | O | . |
| *Ballota nigra** | Lamiaceae | *.* | *.* | O | O | O | O |
| *Barbarea vulgaris** | Brassicaceae** | *.* | *.* | *.* | O | O | O |
| *Dipsacus fullonum** | Caprifoliaceae | *.* | *.* | *.* | O | O | O |
|  |  |  |  |  |  |  |  |
| *Cichorium intybus** | Asteraceae (Cichorioideae)** | *.* | *.* | *.* | *.* | O | O |
| *Potentilla reptans** | Rosaceae | *.* | *.* | *.* | *.* | O | O |
| *Reseda lutea** | Resedaceae | *.* | *.* | *.* | *.* | O | O |
|  |  |  |  |  |  |  |  |
| *Bunias orientalis** | Brassicaceae** | *.* | *.* | *.* | *.* | *.* | O |
| *Cardaria draba** | Brassicaceae** | *.* | *.* | *.* | *.* | *.* | O |
| *Leonurus cardiaca** | Lamiaceae | *.* | *.* | *.* | *.* | *.* | O |
| *Marrubium vulgare** | Lamiaceae | *.* | *.* | *.* | *.* | *.* | O |
| *Muscari comosum** | Liliaceae | *.* | *.* | *.* | *.* | *.* | O |
|  | | | | | | | |
| ANNUALS | | | | | | | |
| *Atriplex patula** | Amaranthaceae** | O | O | O | O | O | O |
| *Bidens cernua* | Asteraceae (Asteroideae)** | O | O | O | O | O | O |
| *Centaurea cyanus* | Asteraceae | O/. | O | O | O | . | O |
| *Cerastium arvense* | Caryophyllaceae** | O | . | . | O | . | O |
| *Chenopodium album** | Amaranthaceae** | O | O | O | O | O | O |
| *Euphorbia peplus** | Euphorbiaceae | O | . | . | . | O | O |
| *Fallopia convolvulus* | Polygonaceae | O/. | O | O | O | O | O |
| *Galeopsis tetrahit** | Lamiaceae | O | O | O | O | O | O |
| *Persicaria hydropiper** | Polygonaceae | O | O | O | O | O | O |
| *Persicaria lapathifolia** | Polygonaceae | O | O | O | O | O | O |
| *Persicaria maculosa* | Polygonaceae | O/. | O | O | O | O | O |
| *Persicaria minor** | Polygonaceae | O | O | O | O | O | O |
| *Persicaria mitis** | Polygonaceae | O | O | . | O | O | O |
| *Polygonum aviculare* | Polygonaceae | O/. | O | O | O | O | O |
| *Rumex maritimus* (other)* | Polygonaceae | O | . | . | O | O | O |
| *Stellaria media* (other) | Caryophyllaceae** | O | O | O | O | O | O |
| *Viola arvensis* | Violaceae | O | O | O | O | O | O |
|  |  |  |  |  |  |  |  |
| *Aethusa cynapium** | Apiaceae** | . | O | O | O | O | O |
| *Agrostemma githago* | Caryophyllaceae** | . | O | O | O | O | O |
| *Anagallis arvensis** | Primulaceae | . | O | O | O | O | O |
| *Aphanes arvensis** | Rosaceae | . | O | . | O | O | O |
| *Arabidopsis thaliana** | Brassicaceae** | . | O | . | . | . | . |
| *Arenaria serpyllifolia* | Caryophyllaceae** | . | O | O | O | O | O |
| *Avena fatua* | Poaceae** | . | O | O | O | O | O |
| *Bromus arvensis* | Poaceae** | . | O | O | O | O | O |
| *Bromus secalinus* | Poaceae** | . | O | O | O | O | O |
| *Bromus sterilis* | Poaceae** | . | O | O | O | O | O |
| *Capsella bursa-pastoris** | Brassicaceae** | . | O | O | O | O | O |
| *Chenopodium ficifolium** | Amaranthaceae** | . | O | O | O | O | O |
| *Chenopodium polyspermum** | Amaranthaceae** | . | O | O | O | O | O |
| *Convolvulus arvensis** | Convolvulaceae | . | O | O | O | O | O |
| *Descurainia sophia** | Brassicaceae** | . | O | . | O | O | O |
| *Digitaria ischaemum** | Poaceae** | . | O | O | O | O | O |
| *Echinochloa crus-galli** | Poaceae** | . | O | O | O | O | O |
| *Fumaria officinalis** | Papaveraceae | . | O | O | O | O | O |
| *Galium aparine** | Rubiaceae | . | O | O | O | O | O |
| *Galium spurium** | Rubiaceae | . | O | O | O | O | O |
| *Geranium dissectum** | Geraniaceae | . | O | O | . | O | O |
| *Hordeum murinum* | Poaceae** | . | O | . | O | . | O |
| *Hyoscyamus niger** | Solanaceae | . | O | O | O | O | O |
| *Lamium purpureum** | Lamiaceae | . | O | O | O | O | O |
| *Lithospermum arvense** | Boraginaceae | . | O | O | O | O | O |
| *Lolium temulentum** | Poaceae** | . | O | O | O | O | O |
| *Malva pusilla** | Malvaceae | . | O | O | O | O | O |
| *Matricaria recutita* | Asteraceae (Asteroideae)** | . | O | . | . | O | O |
| *Myosotis arvensis** | Boraginaceae | . | O | O | O | O | O |
| *Papaver argemone* * | Papaveraceae | . | O | O | O | O | O |
| *Papaver rhoeas** | Papaveraceae | . | O | O | O | O | O |
| *Raphanus raphanistrum* * | Brassicaceae** | . | O | O | O | O | O |
| *Reseda luteola** | Resedaceae | . | O | . | . | O | O |
| *Scleranthus annuus* | Caryophyllaceae** | . | O | O | O | O | O |
| *Setaria pumila** | Poaceae** | . | O |  | O | O | O |
| *Setaria viridis** | Poaceae** | . | O | O | O | O | O |
| *Silene latifolia* | Caryophyllaceae** | . | O | O | O | O | O |
| *Sinapis arvensis* | Brassicaceae** | . | O | O | O | O | O |
| *Sisymbrium officinale** | Brassicaceae** | . | O | O | O | O | O |
| *Solanum nigrum** | Solanaceae | . | O | O | O | O | O |
| *Sonchus oleraceus** | Asteraceae (Cichorioideae)** | . | O | O | O | O | O |
| *Spergula arvensis** | Caryophyllaceae** | . | O | O | O | O | O |
| *Thlaspi arvense** | Brassicaceae | . | O | O | O | O | O |
| *Torilis japonica* | Apiaceae** | . | O | O | O | O | O |
| *Trifolium arvense* | Fabaceae | . | O | O | O | O | O |
| *Urtica urens* | Urticaceae | . | O | O | O | O | O |
| *Valerianella dentata* | Caprifoliaceae | . | O | O | O | O | O |
| *Valerianella locusta* | Caprifoliaceae | . | O | O | O | O | O |
| *Veronica hederifolia* | Plantaginaceae | . | O | O | O | O | O |
| *Vicia hirsuta* | Fabaceae | . | O | O | O | O | O |
| *Vicia tetrasperma* | Fabaceae | . | O | O | O | O | O |
|  |  |  |  |  |  |  |  |
| *Adonis aestivalis* | Ranunculaceae*** | . | . | O | . | . | O |
| *Anchusa arvensis* | Boraginaceae | . | . | O | . | O | O |
| *Anthemis arvensis** | Asteraceae (Asteroideae)** | . | . | O | O | O | O |
| *Bromus tectorum* | Poaceae** | . | . | O | O | O | O |
| *Conringia orientalis** | Brassicaceae** | . | . | O | . | . | O |
| *Datura stramonium** | Solanaceae | . | . | O | . | . | O |
| *Erodium cicutarium** | Geraniaceae | . | . | O | O | O | O |
| *Euphorbia helioscopia** | Euphorbiaceae | . | . | O | O | O | O |
| *Euphorbia platyphyllos** | Euphorbiaceae | . | . | O | . | . | O |
| *Geranium columbinum** | Geraniaceae | . | . | O | . | . | . |
| *Geranium pusillum** | Geraniaceae | . | . | O | . | O | O |
| *Heliotropium europaeum** | Boraginaceae | . | . | O | . | . | O |
| *Mercurialis annua** | Euphorbiaceae | . | . | O | . | O | O |
| *Orlaya grandiflora* | Apiaceae** | . | . | O | . | O | O |
| *Rapistrum perenne** | Brassicaceae** | . | . | O | . | . | . |
| *Anthemis cotula* | Asteraceae (Asteroideae)** | . | . | . | O | O | O |
| *Digitaria sanguinalis** | Poaceae** | . | . | . | O | O | O |
| *Euphorbia exigua** | Euphorbiaceae | . | . | . | O | O | O |
| *Fumaria vaillantii** | Papaveraceae | . | . | . | O | . | O |
| *Lamium amplexicaule** | Lamiaceae | . | . | . | O | O | O |
| *Malva neglecta** | Malvaceae | . | . | . | O | . | O |
| *Odontites vulgaris** | Orobanchaceae | . | . | . | O | O | O |
| *Odontites verna** | Orobanchaceae | . | . | . | O | O | O |
| *Plantago arenaria** | Plantaginaceae | . | . | . | O | . | . |
| *Poa annua** | Poaceae** | . | . | . | O | O | O |
| *Ranunculus arvensis* | Ranunculaceae** | . | . | . | O | O | O |
| *Tripleurospermum perforatum** | Asteraceae (Asteroideae)** | . | . | . | O | O | O |
| *Vicia villosa* | Fabaceae | . | . | . | O | O | O |
| *Anchusa officinalis** | Boraginaceae | . | . | . | . | O | O |
| *Barbarea intermedia** | Brassicaceae** | . | . | . | . | O | . |
| *Legousia speculum-veneris** | Campanulaceae** | . | . | . | . | O | O |
| *Lepidium campestre** | Brassicaceae** | . | . | . | . | O | O |
| *Lepidium ruderale** | Brassicaceae** | . | . | . | . | O | O |
| *Neslia paniculata** | Brassicaceae** | . | . | . | . | O | O |
| *Nigella arvensis* | Ranunculaceae** | . | . | . | . | O | O |
| *Portulaca oleracea** | Portulacaceae | . | . | . | . | O | O |
| *Silene noctiflora other* | Caryophyllaceae** | . | . | . | . | O | O |
| *Xanthium strumarium* | Asteraceae (Asteroideae)** | . | . | . | . | O | O |
| *Cerinthe minor** | Boraginaceae | . | . | . | . | . | O |
| *Cynoglossum officinale** | Boraginaceae | . | . | . | . | . | O |
| *Elsholtzia ciliata** | Lamiaceae | . | . | . | . | . | O |
|  | | | | | | | |
| GRASSES | | | NL | BA | IA | RP | MA |
| *Anthoxanthum odoratum** | Poaceae** | | O | . | O | O | O |
| *Bromus hordeaceus* | Poaceae** | | O | O | O | O | O |
| *Festuca rubra/ovina** | Poaceae** | | O | O | O | O | O |
| *Lolium perenne** | Poaceae** | | O | . | O | O | O |
| *Molinia caerulea** | Poaceae** | | O | . | . | O | O |
| *Phleum pratense** | Poaceae** | | O | O. | O | O | . |
| *Poa pratensis** | Poaceae** | | O | O | O | O | O |
| *Poa trivialis** | Poaceae** | | O | O | O | O | O |
| *Holcus lanatus** | Poaceae** | | *.* | O | O | O | O |
| *Cynosurus cristatus** | Poaceae** | | *.* | *.* | O | O | O |
| *Dactylis glomerata** | Poaceae** | | *.* | *.* | O | O | O |
| *Helictotrichon pratensis** | Poaceae** | | *.* | *.* | O | . | . |
| *Briza media** | Poaceae** | | *.* | *.* | *.* | O | . |
| *Bromus erectus* | Poaceae** | | *.* | *.* | *.* | O | O |
| *Festuca pratensis** | Poaceae** | | *.* | *.* | *.* | O | O |
| *Holcus mollis** | Poaceae** | | *.* | *.* | *.* | O | . |
| *Alopecurus pratensis** | Poaceae** | | *.* | *.* | *.* | *.* | O |
| *Arrhenatherum elatius** | Poaceae** | | *.* | *.* | *.* | *.* | O |
| *Trisetum flavescens** | Poaceae** | | *.* |  | *.* | *.* | O |
|  | | | | | | | |
| OTHER HERBS | | | | | | | |
| *Achillea millefolium* | Asteraceae (Asteroideae)** | | O | . | O | O | O |
| *Cerastium holosteoides** | Caryophyllaceae** | | O | O | O | O | O |
| *Daucus carota* | Apiaceae** | | O | O | O | O | O |
| *Heracleum sphondylium* | Apiaceae** | | O | . | . | O | O |
| *Knautia arvensis* | Caprifoliaceae | | O | . | O | O | O |
| *Leontodon* cf. *autumnalis** | Asteraceae (Cichorioideae)** | | O | . | . | O | O |
| *Leucanthemum vulgare** | Asteraceae (Asteroideae)** | | O | . | O | O | O |
| *Lychnis flos-cuculi** | Caryophyllaceae** | | O | O | O | O | O |
| *Pastinaca sativa** | Apiaceae** | | O | O | . | O | O |
| *Prunella vulgaris** | Lamiaceae | | O | O | O | O | O |
| *Ranunculus acris* | Ranunculaceae** | | O | O | O | O | O |
| *Rumex* cf. *acetosa* | Polygonaceae | | O | O | . | O | O |
| *Silaum silaus* | Apiaceae** | | O | . | . | O | O |
| *Stellaria graminea* | Caryophyllaceae** | | O | O | O | O | O |
| *Trifolium dubium** | Fabaceae | | O | . | O | O | . |
| *Trifolium repens* | Fabaceae | | O | O | O | O | O |
| *Galium mollugo* | Rubiaceae | | . | O | O | O | O |
| *Lathyrus pratensis* | Fabaceae | | . | O | . | . | . |
| *Linum catharticum* | Linaceae | | . | O | O | O | O |
| *Medicago lupulina** | Fabaceae | | . | O | O | O | O |
| *Pimpinella saxifraga* (major)* | Apiaceae** | | . | O | . | O | . |
| *Ranunculus bulbosus* (other) | Ranunculaceae** | | . | O | . | O | O |
| *Scabiosa columbaria* | Caprifoliaceae | | . | O | . | O | O |
| *Caltha palustris* | Ranunculaceae** | | . | . | O | O | O |
| *Centaurea jacea (other)* | Asteraceae | | . | . | O | O | . |
| *Hypochaeris radicata** | Asteraceae (Cichorioideae)** | | . | . | O | O | O |
| *Pimpinella major* | Apiaceae** | | . | . | O | . | O |
| *Succisa pratensis* | Caprifoliaceae | | . | . | O | . | . |
| *Trifolium campestre (other)* | Fabaceae | | . | . | O | O | O |
| *Trifolium pratense* | Fabaceae | | . | . | O | O | O |
| *Veronica chamaedrys** | Plantaginaceae | | . | . | O | O | . |
| *Anthriscus sylvestris* | Apiaceae** | | . | . | . | O | O |
| *Carum carvi* | Apiaceae** | | . | . | . | O | O |
| *Galium verum* * | Rubiaceae | | . | . | . | O | O |
| *Salvia pratensis** | Lamiaceae | | . | . | . | O | . |
| *Senecio jacobaea* | Asteraceae (Asteroideae)** | | . | . | . | O | O |
| *Tragopogon pratensis* * | Asteraceae (Cichorioideae)** | | . | . | . | O | . |
| *Bellis perennis* * | Asteraceae (Asteroideae)** | | . | . | . | . | O |
|  | | | | | |  |  |
| ADVENTIVES | | | | | |  |  |
| *Ambrosia* | Asteraceae (Asteroideae)** | | T | | |  |  |
| APOPHYTHES | | | | | |  |  |
| *Rumex obtusifolius* | Polygonaceae | | H | | |  |  |
| Melampyrum (pratense) | Orobanchaceae | | T | | |  |  |
| *Pteridium aquilinum* | Dennstaedtiaceae | | G | | |  |  |
| *Polypodium vulgare* | Polypodiaceae | | Ch | | |  |  |
| *Calluna vulgaris* | Ericaceae | | Ch | | |  |  |
| *Juniperus-*type | Cupressaceae | | P | | |  |  |
|  | | | | | |  |  |
| FAMILY ( SUBFAMILY) | | | | | |  |  |
| Poaceae** | | |  | | |  |  |
| Cyperaceae** | | |  | | |  |  |
| Caryophyllaceae** | | |  | | |  |  |
| Brassicaceae** | | |  | | |  |  |
| Ranunculaceae** | | |  | | |  |  |
| Apiaceae** | | |  | | |  |  |
| Chenopodiaceae / Amaranthaceae** | | |  | | |  |  |
| Campanulaceae** | | | H | | |  |  |
| Asteraceae (Asteroideae)** | | |  | | |  |  |
| Asteraceae (Cichorioideae)** | | |  | | |  |  |

**References**

Knörzer K-H (2007) Geschichte der synanthropen Flora im Niederrheingebiet. Philipp von Zabern, Mainz am Rhein

Lang G (1994) Quartäre Vegetationsgeschichte Europas: Methoden und Ergebnisse. Fischer, Jena

Willerding U (1986} Zur Geschichte der Unkräuter Mitteleuropas. Göttinger Schriften zur Vor- und Frühgeschichte 22. Wachholtz, Neumünster
