# Supplementary material for: Influence of taxonomic resolution on the value of anthropogenic pollen indicators
Source: Veg Hist Archaeobot. 2021 May 11;31(1):67–84. doi: 10.1007/s00334-021-00838-x (PMC8738506; doi:10.1007/s00334-021-00838-x)
Supplement: Supplementary file 2 — Supplementary file2 (DOCX 59 KB) [file 334_2021_838_MOESM2_ESM.docx]

*Article title: “*Influence of taxonomic resolution on the value of anthropogenic pollen indicators”

*Journal name:* Vegetation History and Archaeobotany

*Author names:* Mara Deza-Araujo; César Morales-Molino; Marco Conedera; Gianni B. Pezzatti; Salvatore Pasta; Willy Tinner

*Affiliation and e-mail address of the corresponding author*: Insubric Ecosystems, Swiss Federal Institute for Forest, Snow and Landscape Research WSL, Cadenazzo, Switzerland and

Institute of Plant Sciences and Oeschger Centre for Climate Change Research, University of Bern, Bern, Switzerland - mara.deza@wsl.ch

**ESM 2** Pollen taxonomy used in this study, based on the list of accepted pollen types (HL0) and the taxonomic harmonization in two hierarchical levels (HL1 and HL2) of the European Pollen Database (EPD; Giesecke et al. 2019). The first two columns refer to the names of the pollen types used in this study and include synonyms, but do not constitute the EPD accepted names.(*) pollen types that were not included as anthropogenic pollen indicators in the simulated datasets at that level. The indicative capacity categories were taken from (Chytrý et al. 2008; di Castri et al. 1990; Lang 1994; Sõukand and Kalle 2015).

| **Indicative capacity in Var.** | **Var.**  **(Pollen type synonyms)** | **HL0 (accepted EPD names)** | **HL1** | **HL2** | **Family** |
| --- | --- | --- | --- | --- | --- |
| PI^1^, Car | *Avena*-type | *Avena*-type (PI) | Cerealia-type  (PI) | Poaceae  (SI, APO) | Poaceae |
| PI^1^, Car | Cerealia-type | Cerealia-type (PI) |  |  |  |
| PI^1^, Car | *Hordeum*/*Triticum* |  |  |  |  |
| PI^1^, Car | *Hordeum*-type | *Hordeum*-type (PI) |  |  |  |
|  | *Agropyron*-type* |  |  |  |  |
|  | *Glyceria*-type* |  |  |  |  |
| PI^1^, Car | *Secale cereale* | *Secale cereale* (PI) |  |  |  |
| PI^1^, Car | *Triticum*-type | *Triticum*-type (PI) |  |  |  |
| PI^1^, Car | *Zea mays* | *Zea mays* (PI) |  |  |  |
|  | *Lygeum spartum** | *Lygeum spartum** |  |  |  |
|  | Poaceae* | Poaceae  (SI, APO) | Poaceae  (SI, APO) |  |  |
|  | *Hordelymus** |  |  |  |  |
| SI, Gr, ADV | *Bromus* |  |  |  |  |
|  | *Linum** | *Linum** | *Linum** | *Linum*  (SI, APO) | Linaceae |
| SI, APO, Hb | *Linum catharticum* | *Linum catharticum* (SI) | *Linum catharticum*-type (SI, APO) |  |  |
| SI, APO, Hb | *Linum catharticum*-type | *Linum catharticum*-type (SI) |  |  |  |
|  | *Linum austriacum*-type* | *Linum austriacum*-type* |  |  |  |
|  | *Linum usitatissimum/L. bienne** | *Linum usitatissimum/L. bienne* (PI) | *Linum usitatissimum/L. bienne* (PI) |  |  |
| PI^1^, Oil-f | *Linum usitatissimum*-type |  |  |  |  |
| PI^1^, Car | *Fagopyrum* | *Fagopyrum* (PI) | *Fagopyrum* (PI) | *Fagopyrum* (PI) | Polygonaceae |
| PI^1^, Pro | *Vicia faba* | *Vicia faba* (PI) | *Vicia*-type (PI) | *Lathyrus /Vicia*-type  (SI, APO) | Fabaceae |
|  | *Vicia*-type* | *Vicia*-type |  |  |  |
|  | *Vicia cracca*-type* |  |  |  |  |
| PI^1^, Pro | *Pisum sativum* | *Pisum sativum* (PI) |  |  |  |
|  | *Lathyrus/Vicia*-type* | *Lathyrus/Vicia*-type* | *Lathyrus/Vicia*-type* |  |  |
|  | *Humulus lupulus** | *Humulus lupulus** | *Cannabis/ Humulus*  (SI, APO) | *Cannabis/ Humulus*  (SI, APO) | Cannabaceae |
| PI^1^, Oil-f | *Cannabis sativa* | *Cannabis sativa* (PI) |  |  |  |
|  | *Cannabis/ Humulus** | *Cannabis/ Humulus** |  |  |  |
| SI, ADV-An | *Centaurea cyanus*-type | *Centaurea cyanus*-type  (SI, ADV) | *Centaurea cyanus*-type  (SI, APO) | *Centaurea cyanus*-type  (SI, APO) | Asteraceae |
|  | *Centaurea montana*-type* | *Centaurea montana*-type* |  |  |  |
|  | *Polygonum*-type (tricolporate)* | *Polygonum*-type (tricolporate)* | *Polygonum*-type (tricolporate)* | Polygonum- type (tricolporate) (SI, APO) | Polygonaceae |
|  | *Polygonum*-type* |  |  |  |  |
| SI, ADV-An | *Polygonum aviculare*-type | *Polygonum aviculare*-type  (SI, ADV) | *Polygonum aviculare*-type  (SI, ADV) |  |  |
|  | *Bistorta officinalis*-type* | *Bistorta officinalis*-type* | *Bistorta officinalis*-type* |  |  |
|  | *Bistorta*-type* |  |  |  |  |
|  | *Bistorta vivipara** | *Bistorta vivipara** |  |  |  |
| SI, ADV-An | *Fallopia* | *Fallopia* (SI, ADV) | *Fallopia* (SI, ADV) |  |  |
|  | Polygonaceae* | Polygonaceae* | Polygonaceae* | Polygonaceae* |  |
| SI, NEO-An, ADV | *Spergula*-type* | *Spergula*-type  (SI, ADV) | *Spergula*-type  (SI, ADV) | Spergula-type (SI, ADV) | Caryophyllaceae |
| SI, NEO-An, ADV | *Spergula arvensis*-type |  |  |  |  |
|  | *Cerastium*-type* | *Cerastium*-type (SI, APO) | *Cerastium*-type (SI, APO) | Caryophyllaceae (periporate excl. Paronychioideae)  (SI, APO) |  |
| SI, APO-An | *Cerastium arvense* (SI, APO) |  |  |  |  |
|  | *Cerastium cerastioides*-type* |  |  |  |  |
|  | *Cerastium fontanum** |  |  |  |  |
|  | *Stellaria* cf. *S. holostea** |  |  |  |  |
|  | Caryophyllaceae* | Caryophyllaceae (periporate excl. Paronychioideae)* | Caryophyllaceae (periporate excl. Paronychioideae)* |  |  |
|  | Caryophyllaceae (periporate excl. Paronychioideae)* |  |  |  |  |
|  | Caryophyllaceae undifferentiated* |  |  |  |  |
|  | Caryophyllaceae subfam. Silenoideae-type* |  |  |  |  |
|  | *Sagina** | *Sagina** | *Sagina** |  |  |
| SI, ADV | *Scleranthus*  (SI, ADV) | *Scleranthus*  (SI, ADV) | *Scleranthus*  (SI, ADV) |  |  |
|  | *Silene dioica*-type* | *Silene dioica*-type* | *Silene*-type  (SI, APO) |  |  |
|  | *Silene vulgaris*-type* | *Silene vulgaris*-type* |  |  |  |
|  | *Silene*-type* | *Silene*-type  (SI, APO) |  |  |  |
|  | *Silene viscaria*-type* |  |  |  |  |
| SI, ADV, NEO-An | *Arenaria*  (SI, ADV) |  |  |  |  |
| SI, APO, NEO- BiP, | *Saponaria*  (SI, APO) | *Saponaria*  (SI, APO) |  |  |  |
|  | *Stellaria holostea** | *Stellaria holostea** | *Stellaria holostea** |  |  |
|  | *Gypsophila repens*-type* | *Gypsophila repens*-type* | *Gypsophila repens*-type* |  |  |
|  | *Gypsophila arrostii** |  |  |  |  |
| SI, ADV, NEO-An | *Agrostemma githago* | *Agrostemma githago* (SI, ADV) | *Agrostemma githago* (SI, ADV) |  |  |
|  | *Dianthus/*  *Petrorhaghia** | *Dianthus/*  *Petrorhaghia** | *Dianthus/*  *Petrorhaghia** |  |  |
|  | *Dianthus*-type* |  |  |  |  |
|  | *Dianthus superbus*-type* |  |  |  |  |
|  | *Moehringia*-type* | *Moehringia*-type* | *Moehringia*-type* |  |  |
|  | *Minuartia*-type* |  |  |  |  |
|  | *Corrigiola litoralis** | *Corrigiola litoralis** | *Corrigiola litoralis** | *Corrigiola litoralis** |  |
|  | *Herniaria** | *Herniaria** | *Herniaria** | Caryophyllaceae subfam. Paronychioideae* |  |
|  | *Herniaria*-type* | *Herniaria*-type* |  |  |  |
|  | *Herniaria glabra*-type* | *Herniaria glabra*-type* |  |  |  |
|  | *Paronychia** | *Paronychia** | *Paronychia** |  |  |
| SI, APO, Hb | *Rumex acetosa*-type | *Rumex acetosa*-type  (SI, APO) | *Rumex acetosa*-type  (SI, APO) | Rumex/Oxyria  (SI, APO) | Polygonaceae |
| SI, Hb, APO- BiP | *Rumex acetosa/R. acetosella*-type |  |  |  |  |
| SI, APO- BiP | *Rumex acetosella*-type | *Rumex acetosella*-type (SI, APO) | *Rumex acetosella*-type (SI, APO) |  |  |
| SI, APO- BiP | *Rumex acetosella* |  |  |  |  |
|  | *Rumex crispus*-type* | *Rumex obtusifolius*-type (SI, APO) | *Rumex obtusifolius*-type (SI, APO) |  |  |
| SI, APO | *Rumex obtusifolius*-type |  |  |  |  |
|  | *Rumex/Oxyria** | *Rumex/Oxyria** | *Rumex/Oxyria** |  |  |
|  | *Rumex** |  |  |  |  |
|  | *Rumex scutatus*-type* |  |  |  |  |
|  | *Rumex alpinus*-type* | *Rumex alpinus*-type* | *Rumex alpinus*-type* |  |  |
|  | *Polygonum* (pericolpate/  periporate)* | *Polygonum* (pericolpate/  periporate)* | *Polygonum* (pericolpate/  periporate)* | *Polygonum* (pericolpate/  periporate)  (SI, APO) |  |
| SI, ADV-An | *Polygonum persicaria*-type | *Persicaria maculosa*-type (SI, ADV) | *Persicaria maculosa*-type (SI, ADV) |  |  |
| SI, ADV-An | *Persicaria maculosa*-type |  |  |  |  |
|  | *Hornungia*-type* | *Hornungia*-type* | Brassicaceae  (SI, APO) | Brassicaceae  (SI, APO) | Brassicaceae |
|  | *Matthiola** | *Matthiola** |  |  |  |
| SI, APO | *Sinapis* | *Draba*-type  (SI, APO) |  |  |  |
|  | *Draba*-type* |  |  |  |  |
|  | *Cardamine** |  |  |  |  |
|  | Brassicaceae* | Brassicaceae |  |  |  |
|  | Asteraceae subf. Asteroideae* | Asteraceae subf. Asteroideae* | Asteraceae subf. Asteroideae* | Asteraceae subf. Asteroideae  (SI, APO) | Asteraceae |
| SI, ADV, NEO-An | *Matricaria*-type | *Matricaria*-type (SI, APO) | *Matricaria*-type (SI, APO) |  |  |
| SI, APO, Gr | *Achillea*-type |  |  |  |  |
| SI, APO | *Anthemis*-type |  |  |  |  |
|  | *Aster*-type* | *Aster*-type  (SI, APO) | *Senecio*-type (SI, APO) |  |  |
|  | *Solidago** |  |  |  |  |
| SI, APO, Hb | *Bellis* |  |  |  |  |
|  | *Eupatorium** |  |  |  |  |
| SI, APO-An | *Bidens*-type | *Bidens*-type  (SI, APO) |  |  |  |
|  | *Arnica montana** |  |  |  |  |
| SI, APO, Gr | *Senecio*-type | *Senecio*-type  (SI, APO) |  |  |  |
|  | *Filago*-type* | *Gnaphalium*-type (SI, APO) |  |  |  |
|  | *Gnaphalium*-type* |  |  |  |  |
| SI, APO | *Erigeron* |  |  |  |  |
|  | *Calendula** | *Calendula** |  |  |  |
|  | *Tussilago*-type* | *Tussilago*-type* |  |  |  |
|  | *Petasites** | *Petasites** |  |  |  |
|  | *Homogyne*-type* | *Homogyne** |  |  |  |
|  | *Ambrosia/*  *Xanthium** | *Ambrosia/*  *Xanthium** | *Ambrosia/*  *Xanthium*  (SI, APO) | *Ambrosia/*  *Xanthium*  (SI, APO) |  |
|  | *Xanthium** | *Xanthium** |  |  |  |
|  | *Xanthium spinosum*-type* |  |  |  |  |
| SI, APO | *Ambrosia* | *Ambrosia*  (SI, APO) |  |  |  |
|  | Asteraceae subf. Cichorioideae* | Asteraceae subf. Cichorioideae* | Asteraceae subf. Cichorioideae  (SI, APO) | Asteraceae subf. Cichorioideae  (SI, APO) |  |
|  | *Scorzonera*-type* |  |  |  |  |
|  | *Scorzonera humilis*-type* | *Scorzonera humilis*-type* |  |  |  |
|  | *Trifolium*-type* | *Trifolium*-type* | *Trifolium*-type  (SI, APO) | *Trifolium*-type  (SI, APO) | Fabaceae |
| SI, APO, Hb | *Trifolium pratense*-type | *Trifolium pratense*-type (SI, APO) |  |  |  |
| SI, APO, Hb | *Trifolium repens*-type | *Trifolium repens*-type (SI, APO) |  |  |  |
|  | *Trifolium alpestre*-type* | *Trifolium alpestre*-type* |  |  |  |
|  | *Trifolium badium*-type* | *Trifolium badium*-type* |  |  |  |
|  | *Trifolium alpinum*-type* | *Trifolium alpinum*-type* |  |  |  |
|  | Ranunculaceae* | Ranunculaceae* | Ranunculaceae* | Ranunculaceae* | Ranunculaceae |
| SI, APO, Hb | *Ranunculus acris*-type | *Ranunculus acris*-type (SI, APO) | *Ranunculus acris*-type (SI, APO) | Ranunculus-type (SI, APO) |  |
|  | *Ranunculus*-type* | *Ranunculus*-type* | *Ranunculus*-type* |  |  |
|  | *Anemone/*  *Hepatica** |  |  |  |  |
|  | *Anemone*  */Pulsatilla** |  |  |  |  |
|  | *Clematis** | *Clematis** | *Clematis** |  |  |
|  | *Ranunculus lingua** | *Ranunculus lingua** | *Ranunculus lingua** |  |  |
|  | *Anemone nemorosa*-type* | *Anemone nemorosa*-type* | *Anemone nemorosa*-type* |  |  |
|  | *Anemone hortensis*-type* | *Anemone*-type* |  |  |  |
|  | *Ranunculus* sect. Batrachium* | *Ranunculus aquatilis*-type* | *Ranunculus aquatilis*-type* |  |  |
|  | *Aconitum** | *Aconitum** | *Aconitum** |  |  |
| SI, APO | *Adonis* | *Adonis* (SI, APO) | *Adonis* (SI, APO) | *Adonis* (SI, APO) |  |
| SI, APO | *Caltha*-type | *Caltha*-type  (SI, APO) | *Caltha*-type  (SI, APO) | *Caltha*-type  (SI, APO) |  |
| SI, ADV, NEO-An | *Nigella* | *Nigella* (SI, ADV) | *Nigella* (SI, ADV) | *Nigella* (SI, ADV) |  |
|  | *Delphinium*-type* | *Consolida*-type* | *Consolida*-type* | *Consolida*-type* |  |
|  | *Consolida*-type* |  |  |  |  |
|  | *Helleborus viridis*-type* | *Helleborus viridis*-type* | *Helleborus viridis*-type* | *Helleborus viridis*-type* |  |
|  | *Thalictrum** | *Thalictrum** | *Thalictrum** | *Thalictrum** |  |
| SI, APO | *Ranunculus arvensis*-type | *Ranunculus arvensis*-type  (SI, APO) | *Ranunculus arvensis*-type  (SI, APO) | *Ranunculus arvensis*-type  (SI, APO) |  |
|  | *Trollius europaeus** | *Trollius europaeus** | *Trollius europaeus** | *Trollius europaeus** |  |
|  | *Plantago** | *Plantago** | *Plantago** | Plantago  (SI, APO) | Plantaginaceae |
|  | *Plantago* undifferentiated* |  |  |  |  |
| SI, ADV, PO- BiP | *Plantago lanceolata*-type | *Plantago lanceolata*-type (SI, ADV) | *Plantago lanceolata*-type (SI, ADV) |  |  |
| SI, ADV- BiP | *Plantago major* | *Plantago major* (SI, ADV) | *Plantago major*-type (SI, ADV) |  |  |
| SI, ADV- BiP | *Plantago major*-type | *Plantago major*-type (SI, ADV) |  |  |  |
| SI, ADV- BiP | *Plantago major/*  *P. media*-type |  |  |  |  |
| SI, ADV- BiP | *Plantago media* | *Plantago media* (SI, ADV) |  |  |  |
| SI, ADV- BiP | *Plantago media*-type |  |  |  |  |
|  | *Plantago maritima*-type* | *Plantago maritima*-type* | *Plantago maritima*-type* |  |  |
|  | *Plantago alpina*-type* |  |  |  |  |
|  | *Plantago tenuiflora** | *Plantago tenuiflora** | *Plantago tenuiflora** |  |  |
|  | *Plantago montana*-type* | *Plantago atrata*-type* | *Plantago atrata*-type* |  |  |
|  | *Plantago atrata*-type* |  |  |  |  |
|  | *Plantago coronopus*-type* | *Plantago coronopus*-type* | *Plantago coronopus*-type* |  |  |
|  | *Cladium mariscus** | *Cladium mariscus** | Cyperaceae  (SI, APO) | Cyperaceae  (SI, APO) | Cyperaceae |
|  | Cyperaceae* | Cyperaceae* |  |  |  |
|  | *Cyperus** | *Cyperus** |  |  |  |
|  | *Rhynchospora** | *Rhynchospora** |  |  |  |
|  | *Rhynchospora alba** |  |  |  |  |
|  | *Apium** | *Apium** | *Apium** | Apiaceae  (SI, APO) | Apiaceae |
|  | *Bupleurum** | *Bupleurum** | *Bupleurum** |  |  |
| SI, APO, Hb | *Heracleum* | *Heracleum*  (SI, APO) | *Heracleum*  (SI, APO) |  |  |
| SI, APO, Hb | *Pimpinella major*-type | *Pimpinella major*-type (SI, APO) | *Pimpinella major*-type (SI, APO) |  |  |
|  | *Peucedanum*-type* | *Peucedanum*-type* | *Peucedanum*-typ |  |  |
| SI, APO, Hb | *Anthriscus sylvestris*-type | *Anthriscus sylvestris*-type (SI, APO) | *Anthriscus sylvestris*-type (SI, APO) |  |  |
|  | *Sanicula europaea** | *Sanicula europaea** | *Astrantia*-type* |  |  |
|  | *Astrantia*-type* | *Astrantia*-type* |  |  |  |
|  | *Pleurospermum*-type* | *Pleurospermum*-type* | *Pleurospermum*-type* |  |  |
|  | *Cicuta virosa** | *Cicuta virosa** | *Cicuta virosa** |  |  |
| SI, APO, Hb | *Daucus* | *Daucus* (SI, APO) | *Daucus* (SI, APO) |  |  |
|  | *Oenanthe** | *Oenanthe** | *Oenanthe** |  |  |
|  | *Eryngium** | *Eryngium** | *Eryngium** |  |  |
|  | *Meum*-type* | *Meum*-type* | *Meum*-type* |  |  |
|  | *Chaerophyllum hirsutum*-type | *Chaerophyllum hirsutum*-type* | *Chaerophyllum hirsutum*-type* |  |  |
|  | *Bupleurum*-type* | Apiaceae  (SI, APO) | Apiaceae  (SI, APO) |  |  |
|  | *Daucus*-type* |  |  |  |  |
|  | *Heracleum*-type* |  |  |  |  |
|  | *Apium*-type* |  |  |  |  |
|  | *Chaerophyllum** |  |  |  |  |
| SI, APO, Hb | *Carum*-type |  |  |  |  |
|  | Apiaceae* |  |  |  |  |
|  | *Smyrnium*-type* |  |  |  |  |
|  | *Ammi*-type* |  |  |  |  |
| SI, ADV, NEO-An | *Torilis japonica* | *Torilis japonica* (SI, ADV) | *Torilis japonica* (SI, ADV) |  |  |
|  | *Ligusticum mutellina** | *Ligusticum mutellina** | *Ligusticum mutellina** |  |  |
|  | *Seseli*-type* | *Seseli*-type* | *Seseli*-type* |  |  |
|  | *Torilis arvensis** | *Torilis arvensis** | *Falcaria*-type* |  |  |
|  | *Torilis arvensis*-type* | *Falcaria*-type* |  |  |  |
|  | *Falcaria*-type* |  |  |  |  |
|  | *Turgenia latifolia** | *Turgenia latifolia** | *Turgenia latifolia** |  |  |
| SI, APO | *Orlaya* | *Orlaya* (SI, APO) | *Orlaya* (SI, APO) |  |  |
|  | *Sison amomum** | *Sison amomum** | *Sison amomum** |  |  |
|  | Dipsacoideae* | Dipsacoideae* | Dipsacoideae  (SI, APO) | Dipsacoideae (SI, APO) | Caprifoliaceae |
| SI, APO, NEO-BiP | *Dipsacus*-type | *Dipsacus*-type  (SI, APO) |  |  |  |
| SI, APO, Hb | *Knautia* | *Knautia* (SI, APO) | *Knautia* (SI, APO) |  |  |
|  | *Scabiosa** | *Scabiosa** | *Scabiosa*  (SI, APO) |  |  |
| SI, APO, Hb | *Scabiosa columbaria*-type | *Scabiosa columbaria*-type (SI, APO) |  |  |  |
| SI, APO, Hb | *Succisa*-type | *Succisa*-type  (SI, APO) | *Succisa*-type  (SI, APO) |  |  |
|  | Campanulaceae* | Campanulaceae* | Campanulaceae (SI, APO) | Campanulaceae (SI, APO) | Campanulaceae |
| SI, APO | *Jasione montana*-type | *Jasione montana*-type (SI, APO) |  |  |  |
|  | *Campanula/ Phyteuma** | *Campanula/ Phyteuma** | *Campanula/ Phyteuma** |  |  |
|  | *Phyteuma*-type* |  |  |  |  |
|  | *Campanula*-type* |  |  |  |  |
|  | *Arbutus** | *Arbutus** | *Arbutus** | Ericales (tetrads) (SI, APO) | Ericaceae |
| SI, APO | *Calluna vulgaris* | *Calluna vulgaris* (SI, APO) | *Calluna vulgaris* (SI, APO) |  |  |
|  | Ericales (tetrads)* | Ericales (tetrads)* | Ericales (tetrads) (SI, APO)* |  |  |
|  | *Erica** | *Erica** |  |  |  |
|  | *Rhododendron** | *Rhododendron* (SI, APO)* |  |  |  |
|  | *Vaccinium** | *Vaccinium** |  |  |  |
|  | *Vaccinium*-type* | *Vaccinium*-type* |  |  |  |
|  | *Erica arborea*-type* | *Erica arborea*-type* |  |  |  |
|  | *Empetrum/ Ledum** | *Empetrum/ Ledum** | *Empetrum/ Ledum** |  |  |
|  | *Juniperus*-type* | *Juniperus*-type* | *Juniperus*-type (SI, APO) | *Juniperus*-type (SI, APO) | Cupressaceae |
| SI, APO | *Juniperus communis*-type | *Juniperus communis*-type (SI, APO) |  |  |  |
| SI, APO | *Melampyrum* | *Melampyrum*  (SI, APO) | *Melampyrum*  (SI, APO) | *Melampyrum*  (SI, APO) | Orobanchaceae |
| SI, APO | *Pteridium aquilinum* | *Pteridium aquilinum*  (SI, APO) | *Pteridium aquilinum*  (SI, APO) | *Pteridium aquilinum*  (SI, APO) | Dennstaedtiaceae |
| SI, APO | *Polypodium* | *Polypodium*  (SI, APO) | *Polypodium*  (SI, APO) | *Polypodium*  (SI, APO) | Polypodiaceae |
|  | *Atriplex** | Amaranthaceae (SI, APO) | Amaranthaceae (SI, APO) | Amaranthaceae (SI, APO) | Amaranthaceae |
|  | *Salsola*-type* |  |  |  |  |
|  | Amaranthaceae* |  |  |  |  |
| SI, ADV, NEO | *Amaranthus*-type |  |  |  |  |
|  | *Parietaria** | *Parietaria** | *Parietaria** | Urticaceae/  Moraceae  (SI, APO) | Urticaceae/  Moraceae |
| SI, APO | *Urtica* | *Urtica*  (SI, APO) | *Urtica*  (SI, APO) |  |  |
| SI, APO | *Urtica membranacea* |  |  |  |  |
| SI, APO-BiP | *Urtica dioica*-type | *Urtica dioica*-type (SI, APO) |  |  |  |
|  | Urticaceae* | Urticaceae* | Urticaceae* |  |  |
|  | *Morus** | *Morus** | *Morus** |  |  |
|  | *Morus alba** | *Morus alba** |  |  |  |
|  | *Urtica pilulifera** | *Urtica pilulifera** | *Urtica pilulifera** |  |  |
|  | Urticaceae/  Moraceae* | Urticaceae/  Moraceae* | Urticaceae/  Moraceae* |  |  |
| PI^2^, Tr | *Ficus carica* | *Ficus carica* (PI) | *Ficus carica* (PI) | *Ficus carica* (PI) | Moraceae |
| SI, APO | *Artemisia* | *Artemisia*  (SI, APO) | *Artemisia*  (SI, APO) | *Artemisia*  (SI, APO) | Asteraceae |
|  | Oleaceae* | Oleaceae (PI) | Oleaceae (PI) | Oleaceae (PI) | Oleaceae |
| PI^2^, NEO-TS | *Fraxinus/Phillyrea* |  |  |  |  |
| PI^2^, Tr | *Olea europaea* | *Olea europaea* (PI) | *Olea europaea* (PI) | *Olea europaea* (PI) |  |
|  | *Juglans** | *Juglans* (PI) | *Juglans* (PI) | *Juglans* (PI) | Juglandaceae |
| PI^2^, Tr | *Juglans regia* | *Juglans regia* (PI) |  |  |  |
| PI^2^, Tr | *Castanea sativa* | *Castanea sativa* (PI) | *Castanea sativa* (PI) | *Castanea sativa* (PI) | Fagaceae |
| PI^2^, Tr | *Vitis* | *Vitis* (PI) | *Vitis* (PI) | *Vitis* (PI) | Vitaceae |
| PI^1^, NEO-TS | *Eucalyptus* | *Eucalyptus* (PI) | Myrtaceae (PI) | Myrtaceae (PI) | Myrtaceae |
|  | Myrtaceae | Myrtaceae* |  |  |  |
|  | *Myrtus communis** |  |  |  |  |
| PI^2^, Tr | *Pistacia* | *Pistacia* (PI) | *Pistacia* (PI) | *Pistacia* (PI) | Anacardiaceae |

PI^1^ =primary indicators (crops) non-native to Europe; PI^2^ =primary indicators native to Europe. Car = carbohydrate plants; Pro= protein plants; Oil-f= oil and fibber plants; Tr= fruit trees and nuts. SI= secondary indicators (weeds): ADV= adventives; APO= apophytes; NEO= neophytes; BiP= biennials and perennials; An=annuals. Grassland species: Gr=grasses; Hb=other herbs. NEO-TS= cultivated neophytes (trees and shrubs)

**References**

Chytrý M, Maskell LC, Pino J et al (2008) Habitat invasions by alien plants: a quantitative comparison among Mediterranean, subcontinental and oceanic regions of Europe. J Appl Ecol 45:448-458. https://doi.org/10.1111/j.1365-2664.2007.01398.x

Di Castri F, Hansen AJ, Debussche M (eds) (1990) Biological invasions in Europe and the Mediterranean Basin. Monographiae Biologicae 65. Kluwer, Dordrecht. https://doi.org/10.1007/978-94-009-1876-4

Lang G (1994) Quartäre Vegetationsgeschichte Europas: Methoden und Ergebnisse. Fischer, Jena

Sõukand R, Kalle R (2015) Emic conceptualization of a ‘wild edible plant’ in Estonia in the second half of the 20th century. Trames-J Humanit Soc 19(1):15−34. https://doi.org/[10.3176/tr.2015.1.02](http://dx.doi.org/10.3176/tr.2015.1.02)
