# Supplementary material for: Influence of taxonomic resolution on the value of anthropogenic pollen indicators
Source: Veg Hist Archaeobot. 2021 May 11;31(1):67–84. doi: 10.1007/s00334-021-00838-x (PMC8738506; doi:10.1007/s00334-021-00838-x)

*Article title: “*Influence of taxonomic resolution on the value of anthropogenic pollen indicators”

*Journal name:* Vegetation History and Archaeobotany

*Author names:* Mara Deza-Araujo; César Morales-Molino; Marco Conedera; Gianni B. Pezzatti; Salvatore Pasta; Willy Tinner

*Affiliation and e-mail address of the corresponding author*: Insubric Ecosystems, Swiss Federal Institute for Forest, Snow and Landscape Research WSL, Cadenazzo, Switzerland and

Institute of Plant Sciences and Oeschger Centre for Climate Change Research, University of Bern, Bern, Switzerland - mara.deza@wsl.ch

**ESM 3** Change point analysis of the pollen percentage of human indicators at HL0, HL1 and HL2 for each study site using the binary segmentation approach. 1= Egelsee (Menzingen), 2= Burgäschisee, 3= Soppensee, 4= Moossee, 5= Bachalpsee; 6= Lej da San Murezzan, 7= Lej da Champfèr, 8= Lengi Egga, 9= Gouillé Rion, 10= Lago di Origlio, 11= Lago di Muzzano, 12= Lago Piccolo di Avigliana, 13= Pavullo nel Frignano, 14= Lago del Greppo, 15=Lago dell'Accesa, 16=Gorgo Basso. Black solid lines represent pollen percentages, red dashed lines represent detected change points (constrained to a maximum of 4 for each sequence). Note that y-axes (pollen percentage) have different scales


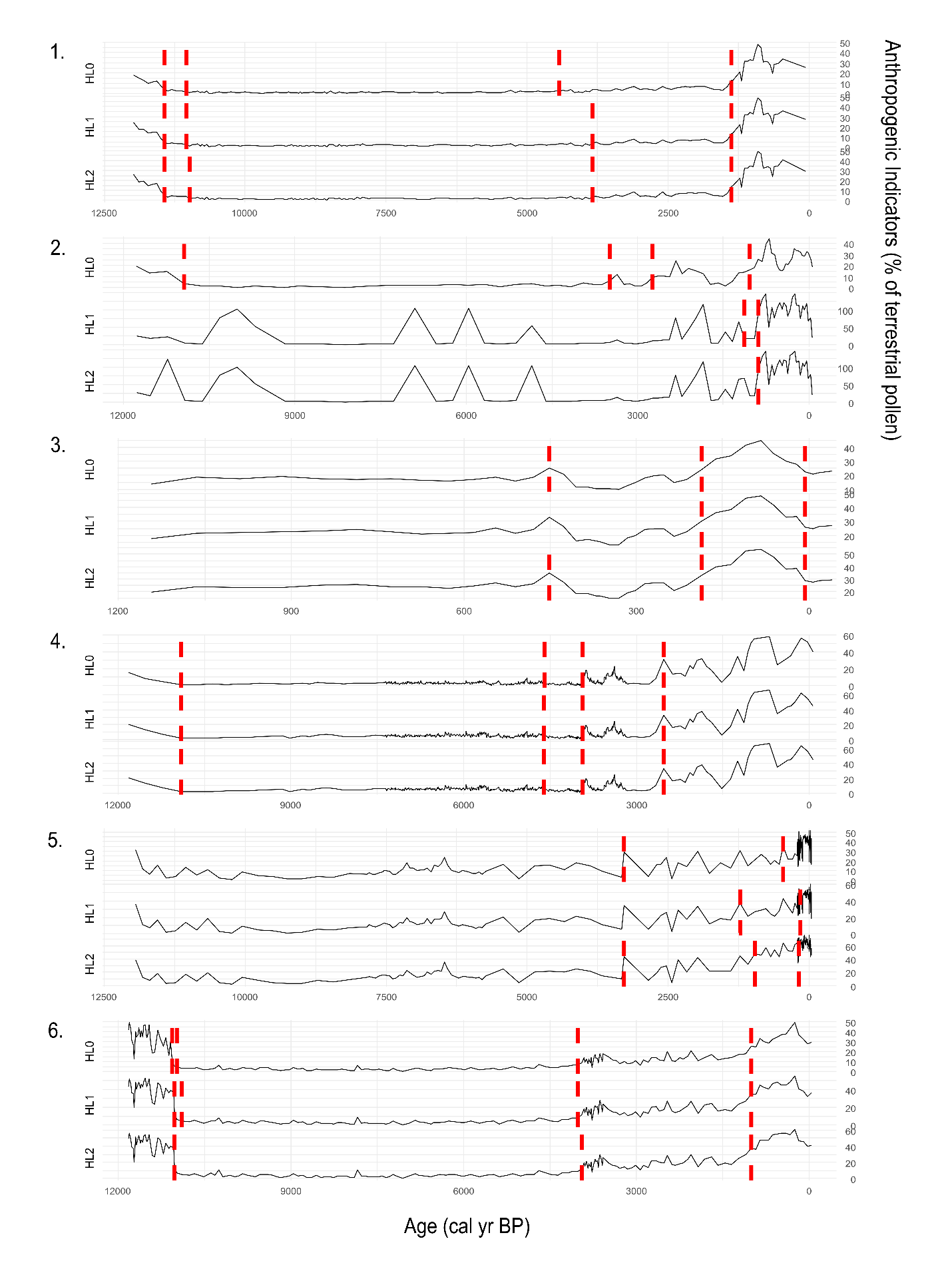


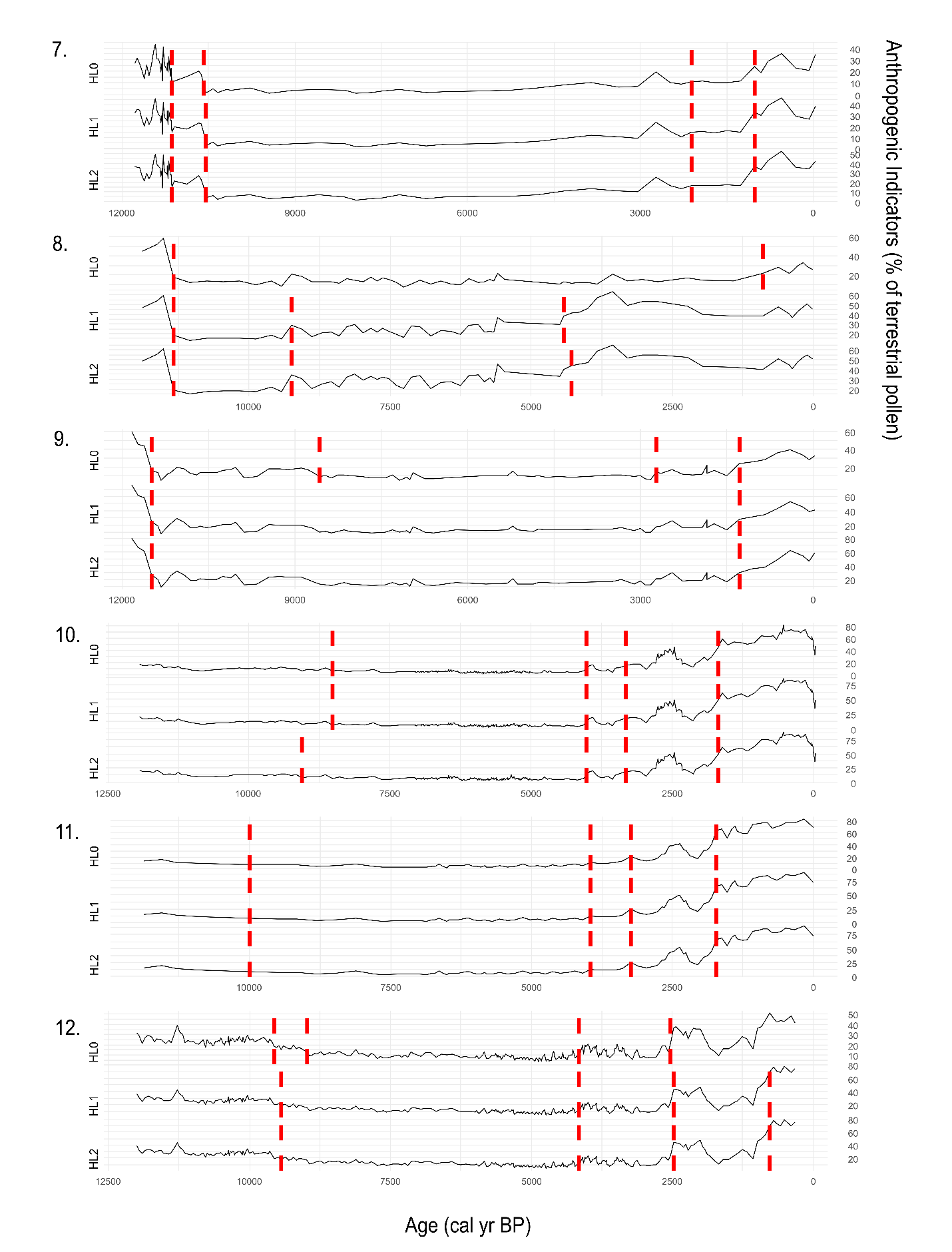


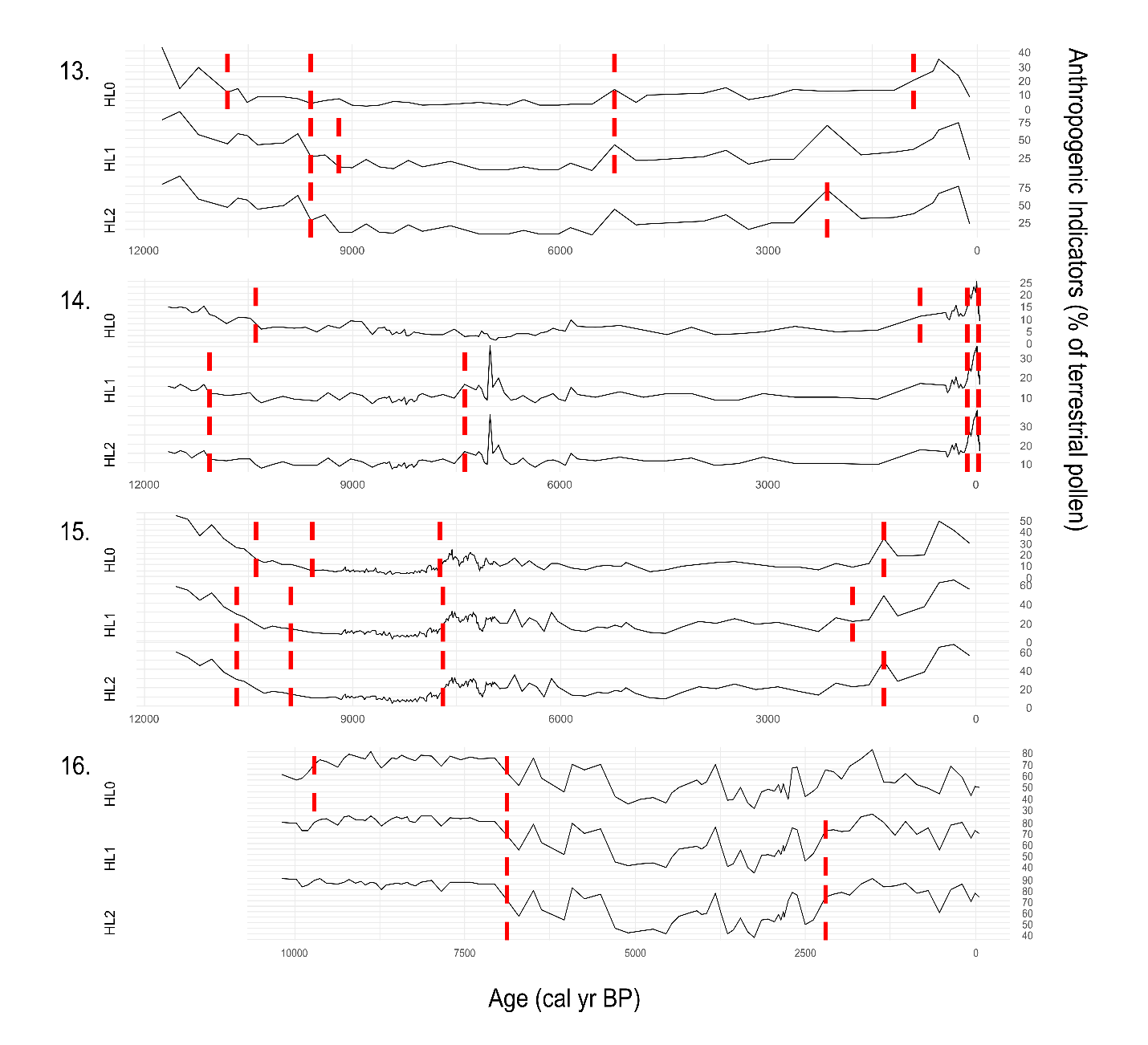

Supplement: Supplementary file 3 — Supplementary file3 (DOCX 600 KB) [file 334_2021_838_MOESM3_ESM.docx]
